# Supplementary material for: Covalent organic frameworks for direct photosynthesis of hydrogen peroxide from water, air and sunlight
Source: Nat Commun. 2023 Jul 19;14:4344. doi: 10.1038/s41467-023-40007-4 (PMC10356944; doi:10.1038/s41467-023-40007-4)
Supplement: Supplementary file 1 — Supplementary Information [file 41467_2023_40007_MOESM1_ESM.pdf]

Supporting Information

Covalent organic frameworks for direct photosynthesis of hydrogen peroxide from water, air and sunlight

Fuyang Liu[1,2,3,7], Peng Zhou[4,7], Yanghui Hou[1,2,3], Hao Tan[5], Yin Liang[5], Jialiang Liang[6], Qing Zhang[5], Shaojun Guo[5], Meiping Tong [1,2,3*], Jinren Ni[1,2,3]

[1] College of Environmental Sciences and Engineering, Peking University, Beijing, 100871, P. R. China.
[2] The Key Laboratory of Water and Sediment Sciences (Ministry of Education), Peking University, Beijing, 100871, P. R. China.
[3] State Environmental Protection Key Laboratory of All Material Fluxes in River Ecosystems, Peking University, Beijing, 100871, P. R. China.
[4] School of Environment and Energy, Peking University Shenzhen Graduate School, Shenzhen, Guangdong, 518055, P. R. China.
[5] School of Materials Science and Engineering, Peking University, Beijing, 100871, P. R. China
[6] College of Environment and Ecology, Chongqing University, Chongqing, 400045, P. R. China
[7] These authors contributed equally: Fuyang Liu, Peng Zhou.
[*]Corresponding author: E-mail address: tongmeiping@pku.edu.cn.

Number of pages: 61
Number of tables: 3
Number of figures: 48

Table of Contents

Index	Caption	Page
**Figure S1**	XPS spectra of three COFs.	S8
**Figure S2**	X-ray diffraction (XRD) patterns of three COFs.	S9
**Figure S3**	SEM images of (a) COF-N31, (b) COF-N32 and (c) COF-N33.	S10
**Figure S4**	TEM images of (a) COF-N31, (b) COF-N32 and (c) COF-N33.	S11
**Figure S5**	Charge contribution in (a) COF-N31, (b) COF-N32 and (c) COF-N33 structures.	S12
**Figure S6**	Solid-state electron spin resonance spectra of three COFs under dark conditions.	S13
**Figure S7**	Dipole moment of the fragments in three COFs.	S14
**Figure S8**	Solvatochromic behavior of (a) COF-N31, (b) COF-N32 and (c) COF-N33.	S15
**Figure S9**	Water contact angles (CA) of water droplet on the pressed pellet of three COFs.	S16
**Figure S10**	The water adsorption isotherms of three COFs.	S17
**Figure S11**	HOMO (up), LUMO (down) orbit distribution of three COFs	S18
**Figure S12**	Calculated band structure and density of state (DOS) of three COFs.	S19
**Figure S13**	Electric impedance spectra of three COFs	S20
**Figure S14**	Integrated PL intensity as a function of temperature of three COFs	S21
**Figure S15**	Steady-state PL spectra of three COFs excited at 350 nm.	S22
**Figure S16**	Transient adsorption spectra of three COFs registered at different probe delays.	S23
**Figure S17**	PL decay curves of COF-N31 (a), COF-N32 (b) and COF-N33 (c).	S24
**Figure S18**	ESR spectra of TEMPO in (a) COF-N31 and (c) COF-N33.	S25
**Figure S19**	Generation of TEMP from TEMPO by the photo electrons in COF-N32.	S26
**Figure S20**	Valence band XPS spectra of three COFs.	S27
**Figure S21**	Schematic illustration of energy band of three COFs.	S28
**Figure S22**	Relationship of H_2O_2 photosynthesis kinetics and intramolecular polarization.	S29
**Figure S23**	Degradation of 1 mM H_2O_2 by three COFs under N_2 atmosphere.	S30
**Figure S24**	H_2O_2 yield by COF-N32 with different dosage of COF-N32.	S31
**Figure S25**	XRD pattern of COF-N32 before and after use.	S32
**Figure S26**	Reusability and structural stability of COF-N31 and COF-N33.	S33

Index	Caption	Page
**Figure S27**	^{18}O isotopic experiment by using $H_2{}^{18}O$ as water source during H_2O_2 production.	S34
**Figure S28**	Photographs of culture media for the disinfection of ARB.	S35
**Figure S29**	In-situ photocatalytic ARB disinfection and diclofenac degradation by COF-N32.	S36
**Figure S30**	Effects of initial solution pH on the H_2O_2 photosynthesis by COF-N32.	S37
**Figure S31**	Photocatalytic H_2O_2 production by COF-N32 in real waters under visible light.	S38
**Figure S32**	Photographs of membrane filter reactor and the performance in the filter.	S39
**Figure S33**	Photocatalytic H_2O_2 production by COF-N32 in real waters in a cloudy day.	S40
**Figure S34**	Light intensities of natural solar irradiation during H_2O_2 production in reactor.	S41
**Figure S35**	H_2O_2 yield in real waters by COF-N32 in membrane reactor under natural sunlight.	S42
**Figure S36**	Immobilization of COF-N32 onto ITO glass for H_2O_2 production.	S43
**Figure S37**	Effects of scavengers on the H_2O_2 photosynthesis kinetics.	S44
**Figure S38**	RRDE curves of COF-N32 and average number of transferred electrons.	S45
**Figure S39**	In-situ ESR spectra of DMPO-$\cdot O_2^-$ for three COFs under visible light irradiation.	S46
**Figure S40**	Thermogravimetric curves of three COFs.	S47
**Figure S41**	N_2 adsorption-desorption isotherms and pore distributions (insets) of (a) COF-N31, (b) COF-N32 and (c) COF-N33. (d) O_2-TPD curves of three COFs.	S48
**Figure S42**	ESR spectra for DMPO-$\cdot$OH in water before and after light irradiation.	S49
**Figure S43**	Time-course in-situ FTIR spectra of COF-N32 with H_2O under dark condition.	S50
**Figure S44**	Peak height for triazine group in the in-situ FTIR spectra.	S51
**Figure S45**	H_2O_2 production in the presence of $NaBrO_3$ (10 mM) under N_2 atmosphere.	S52
**Figure S46**	The oxygen evolution by COF-N32 with $NaBrO_3$ under Ar atmosphere.	S53
**Figure S47**	H_2O_2 yield by COF-N32 and COF-C32 under visible light irradiation.	S54
**Figure S48**	Peak height for adsorbed (a) $\cdot O_2^-$and (b) peroxy species in in-situ FTIR spectra.	S55
**Table S1**	Calculated results for intramolecular polarity of three COFs.	S56
**Table S2**	Calculated parameters for polarity and electrons-holes overlap of three COFs.	S57
**Table S3**	Comparison of photocatalytic H_2O_2 production by reported photocatalysts.	S58

33

Materials and Methods

Materials

1,3,5-triformylphloroglucinol, 1,3,5-triazine-2,4,6-triamine, 4,4',4''-(1,3,5-triazine-2,4,6-triyl)-trianiline, 4,4',4''-(1,3,5-triazine-2,4,6-triyl)tris(([1,1'-biphenyl]-4-amine)) were obtained from Bide pharm Co. (Shanghai, China). NaOH, HCl, and HNO_3 (guaranteed reagents) were purchased from Beijing Chemical Work. All other reagents and solvents were bought from Sigma Aldrich or Sinopharm Chemical Reagent Co., Ltd.

General methods details

Scanning electron microscopy (SEM, Nova NanoSEM 430, FEI, Hillsboro, OR, USA) and transmission electron microscope (TEM, Tecnai F30, FEI, Hillsboro, OR, USA) was used to obtain the morphology of synthesized COFs. Powder X-ray diffraction (XRD, DMAX-2400, Rigaku, Tokyo, Japan) was utilized to reveal the crystalline structure of COFs. Solid state nuclear magnetic resonance (NMR, Bruker-400 AVANCE III, Bruker, Switzerland) and Fourier transform infrared spectroscopy (FT-IR, Nicolet is50, Thermo Fisher Scientific Inc., Waltham, MA, USA) spectra of COFs were obtained to further confirm the organic structure. UV-vis diffuse reflectance spectra (UV-DRS, Lambda 650S spectrophotometer, PerkinElmer, Massachusetts, USA) was utilized to determine the band gap of the fabricated material. X-ray photoelectron spectroscopy (XPS, Axis Ultra, Kratos Analytical Ltd., Manchester, UK) valence band spectra of COFs were obtained to determine the precise valence band position. The N_2 adsorption-desorption isotherms and water adsorption analysis were performed by using an ASAP2010 and 3Flex, respectively (Micromeritics Instrument Ltd., Norcross, GA, USA). The contact angles between COFs and water were measured by using an OCAH200 contact angle analyzer (Dataphysics Co, Germany). The steady state photoluminescence (PL) spectra, time-resolved PL decay curve were measured by FLS980 fluorescence spectrophotometer (Edinburgh, UK) with an excitation wavelength of 365 nm. Temperature-dependent PL spectra were also recorded on FLS980 spectrophotometer with Dewar flask under the temperature of 78-258 K to reveal the binding energy of excitons in COFs. The TA spectroscopy was conducted by using a commercial TA spectrometer (Helios, Ultrafast Systeem, USA) with an excitation source of a femtosecond-pulsed amplifier (Astrella, 80 fs, 1 kHz, Coherent), which was doubled by the Coherent Libra regenerative amplifier (800 nm 80 fs, 1 kHz).

The measurement of H_2O_2

The production of H_2O_2 was measured by iodometry according to the literature[S1]. Specifically, 1 mL of samples was added to the mixture of 1 mL of 0.4 M KI and 1 mL 0.1 M potassium hydrogen phthalate ($C_8H_5KO_4$), which was kept for 1 h. Under acidic conditions, H_2O_2 can react with I^- to generate triiodide anions (I_3^-), which exhibited absorption at 350 nm. Thus, the absorbance at 350 nm by using UV-vis spectroscopy can measure the amount of I_3^-, which can further determine the amount of H_2O_2 produced in each sample.

Details for ESR analysis

Electron spin resonance experiments were conducted by using ESR spectrometer (EMXnano, Bruker, Germany) to detect the solid state radicals, photo-generated electrons (e^-), hydroxyl radicals (·OH) and superoxide ($\cdot O_2^-$). Typically, solid state radicals of COFs could be directly detected in the form of powders. While TEMPO (2,2,6,6-tetramethyl-1-piperidinyloxy) was employed to determine the generation of e^-. DMPO (5, 5-dimethyl-1-pyrroline-N-oxide) was used as spin-trapping agent for the

detection of $\cdot O_2^-$ and $\cdot OH$ in the reaction system in DMSO and water, respectively.

The samples for e^- detection were prepared by mixing 100 μL of catalysts suspension (1 g L^{-1} in water) and 10 μL TEMPO (0.5 mM in water). The samples for $\cdot O_2^-$ detection were prepared by mixing 2 μL DMPO and 200 μL catalysts suspension (1 g L^{-1} in DMSO). The mixture for $\cdot OH$ detection was prepared by mixing 2 μL of DMPO, 0.2 mL of catalysts suspension (1 g L^{-1} in water). The samples prepared above were subsequently loaded in a 1 mm capillary. The intensity of the TEMPO, $\cdot O_2^-$ and $\cdot OH$ signals were then measured under the visible light irradiation, respectively.

Details for in-situ FTIR measurement

In-situ Fourier transform infrared measurement was conducted on an in-situ infrared diffuse reflectance infrared Fourier transform (DRIFT) spectroscopy on a Bruker Tensor II FTIR spectrometer. The photocatalysts were filled into an in-situ IR cell in a chamber. Before the measurement, the chamber was degassed under Ar flow at 393 K for 2 h. The baseline was then obtained at room temperature.

For the investigation of water dissociation, 5 μL of H_2O was added onto the samples, subsequently with the continuous Ar flow through the chamber. The in-situ FTIR spectra were then obtained at specific intervals until the equilibrium was achieved. For the investigation of H_2O_2 photosynthesis, 10 mL of O_2 was then directly injected into the sealed chamber, followed by the visible light irradiation through the window of the chamber. The in-situ FTIR spectra were recorded at specific intervals.

Electrochemical measurement

Photoelectrochemical measurements were performed on a CHI760E electrochemical workstation. A standard three-electrode system was employed with a platinum foil and Ag/AgCl as the counter electrode and the reference electrode, respectively. The working electrodes were prepared as follows. Typically, 10 mg of the COFs were well dispersed in a mixture of ethanol (560 μL) and Nafion (40 μL, 5% v/v) under the ultrasonication for 30 min. Then, 0.1 mL of the suspension was dropwise coated on the indium tin oxide (ITO) glass substrate. 0.2 M Na_2SO_4 aqueous solution was employed as the electrolyte. Electrochemical impedance spectra (EIS) was performed with the frequency ranged from 1×10^6 Hz to 1 Hz with an AC amplitude of 20 mV. Mott-Schottky (M-S) curves were investigated at a frequency of 500 Hz. Rotating ring-disk electrode (RRDE) analysis was conducted in a three-electrode cell by using Pt foil as a counter electrode and using Ag/AgCl as a reference electrode, respectively. The RRDE was consist of a glassy carbon disk and Pt ring. Before the experiment, COF-N32 was dropped onto the glassy carbon disk and then dried.

Determination of SCC efficiency

The photocatalytic reaction was conducted in ultrapure water (2 mL) with COFs (3 mg) in a round-bottomed Pyrex glass flask with magnetic stirring. After O_2 bubbling, the suspension was irradiated by an Xe lamp with light intensity of 100 mW cm^{-2}. Typically, the SCC efficiency was calculated by using the following equation:

SCC efficiency (%) = {[△G for H_2O_2 generation (J mol^{-1})] × [H_2O_2 formed (mol)]}/{[total input ower (W)×[reaction time (s)]}×100% (S1)

where △G= 117 kJ mol^{-1}. In this study, COF-N32 is employed, the irradiated area is 1×10^{-4} m^2, and the light intensity of Xenon lamp is 100 mW cm^{-2}. Thus, the total input power is calculated to be 0.1 W.

The SCC efficiency of COF-N32 in pure water was determined to be 0.31%, which was significantly higher than solar-to-biomass efficiency by plants (~0.1%).

Determination of apparent quantum yield (AQY)

The AQY of COF-N32 at specific wavelength can be calculated by the following equation:

$$\Phi_{AQY}\ (\%) = (\text{the number of } H_2O_2 \text{ molecules} \times 2) / (\text{photon number}) \times 100\% \quad (S2)$$

Analytical methods of UPLC-MS/MS for TEMPO and the reduction product (TEMP)

TEMPO (2,2,6,6-tetramethyl-1-piperidinyloxy) and the reduction product TEMP were detected by the ultra-high-performance liquid chromatography (UHPLC, Dionex UltiMate 3000 Series) coupled with an Orbitrap mass spectrometer (MS, Thermo Scientific, USA) in a positive ion mode ($[M\text{-}H]^+$ ion). After the filtration by 0.22 μm membrane, 5 μL of the samples was injected into the instrument and subsequently separated by a Waters acquity UPLC BEH C18 column (2.1 × 100 mm, 1.7 μm) with the column temperature of 30°C at a flow rate of 0.2 mL min^{-1}. The mobile phase was a mixture of A (methanol) and B (ultrapure water). Gradient elution steps were initially started with A/B 10:90 (v/v), increased linearly to 90:10 (v/v) within 10 min and kept for 2 min, followed by a linear gradient to the initial composition within 0.1 min and kept for another 2.9 min. A selective ion recording (SIR) mode with a dwell time of 200 ms was employed to acquire MS spectra with a scan range of m/z 50-750. The desolvation temperature was set at 350 °C. The spray voltage and cone voltage were 3.5 kV and 40 V, respectively.

Disinfection of antibiotics resistant bacteria (ARB)

Kanamycin-resistant *E. coli*, a model type of ARB, was cultivated in 100 mL Luria Broth growth medium, containing 10 g L^{-1} tryptone, 10 g L^{-1} NaCl, 5 g L^{-1} bacto-yeast extract and 50 mg L^{-1} kanamycin. The flask with the growth media were shaken at 200 revolutions per minute (rpm) in an incubator at 37 °C for 16 h. Bacterial cells were harvested by centrifugation at ~1400 × g for 8 min. The growth media were then discarded after the centrifugation. Subsequently, the bacterial pellets were washed with sterilized physiological saline (0.9% of NaCl solution at pH 7.0) for three times to remove the residual growth medium. The cell precipitates were then re-dispersed in sterilized physiological saline with certain volumes to obtain the bacterial stock suspensions. The viable cell density of the bacterial stock suspension was typically diluted to 5.0×10^9 colony forming unit per milliliter (CFU mL^{-1}).

For typical photocatalytic disinfection experiment, 10 mg of COFs was well dispersed in 49.8 mL sterilized saline with 15 mM NaCl under sonication. Certain amount of ARB stock suspension was added into COFs suspension to set the target viable cell density of 1.0×10^5 CFU mL^{-1}. The suspensions were then magnetically stirring for 5 min prior to the visible light irradiation. During the disinfection process, 0.5 mL bacterial suspension was sampled at given intervals and serially diluted with sterilized deionized water. After that, 0.1 mL of the diluted samples were immediately spread on nutrient agar plates and incubated at the temperature of 37 °C for 24 h.

^{18}O isotopic experiment

$H_2{}^{18}O$ with saturated $^{16}O_2$ was employed to replace water in a sealed reactor for H_2O_2 production by COFs, which was irradiated by Xenon lamp (>420 nm). After H_2O_2 photosynthesis, the reaction suspension was purged by N_2 to remove $^{16}O_2$ in the reactor. Subsequently, the photo-generated H_2O_2

was decomposed into O_2 by adding catalase. The generated O_2 gas was analyzed by a gas chromatography-mass spectrometry (GC-MS, Agilent 7890B-5977B).

Theoretical calculation

The intramolecular polarization of COFs as well as the separation of electrons and holes in COFs was analyzed by using the hybrid b3lyp DFT functions with the basis sets of 6-31G. After the geometry optimization, Gaussian 16 package with Multiwfn was used as wave function analysis tool for the DFT calculations. The intramolecular polarization was calculated by the ratio of dipole moment to the area of the fragments in COFs. The vector of dipole moment was visualized by Multiwfn Ver 3.7 (released on Aug 14, 2020). The molecular polarity index (MPI) and the S_m index (for the description of hole-electron overlap) of the three COFs were also analyzed by using Multiwfn Ver 3.7.

The electronic structure of fabricated COFs was investigated by the Vienna Ab-initio Simulation Package (VASP) using the Perdew-Burke-Ernzerhof (PBE) of the generalized gradient approximation (GGA). The PAW pseudo-potential of N ($2s^2 2p^3$), C ($2s^2 2p^2$), O ($2s^2 2p^4$) and H ($1s^1$) potentials were employed to describe the interaction between valence electrons and the ionic core. The geometry optimization of COFs was conducted by a plane-wave basis with energy cutoff of 400 eV and an energy convergence threshold of 1.0×10^{-4} eV at the gamma point. Based on the geometry optimization, the lattice parameters of the COFs were predicted to be a=11 Å, b=20 Å for COF-N31, a=19 Å, b=33 Å for COF-N32 and a=26 Å, b=45 Å for COF-N33. The vacuum layer between two neighboring layers of COFs was set to 15 Å, which was used to exclude the interaction between neighboring COFs layers. The projected density of state (PDOS) and charge density mappings were calculated with the cutoff energy of 400 eV, energy convergence of 1×10^{-5} eV. The Monkhorst-Pack k-point meshes are 4 × 2 × 4 for COF-N31, 2 × 2 × 4 for COF-N32 and 2 × 2 × 4 for COF-N33. The calculations of Gibbs free energy changes (ΔG) of all reaction steps adopted the known standard hydrogen electrode (SHE) model. The G was calculated by the following formula:

$$G = E + H(T) - TS \quad \text{(S3)}$$

where E, H(T) and S are the electronic free energy, enthalpy and entropy of model at T = 298.15 K, respectively. The highest reaction energy of elemental steps was used as the energy barrier in each pathway.

Reaction equations

$$\text{COF-N32} \rightarrow e^- + h^+ \quad \text{(S4)}$$

$$O_2 + e^- \rightarrow \cdot O_2^- \quad \text{(S5)}$$

$$\cdot O_2^- + e^- + 2H^+ \rightarrow H_2O_2 \quad \text{(S6)}$$

$$H_2O + h^+ \rightarrow {*OH} + H^+ \quad \text{(S7)}$$

$$2 \cdot OH \rightarrow H_2O_2 \quad \text{(S8)}$$

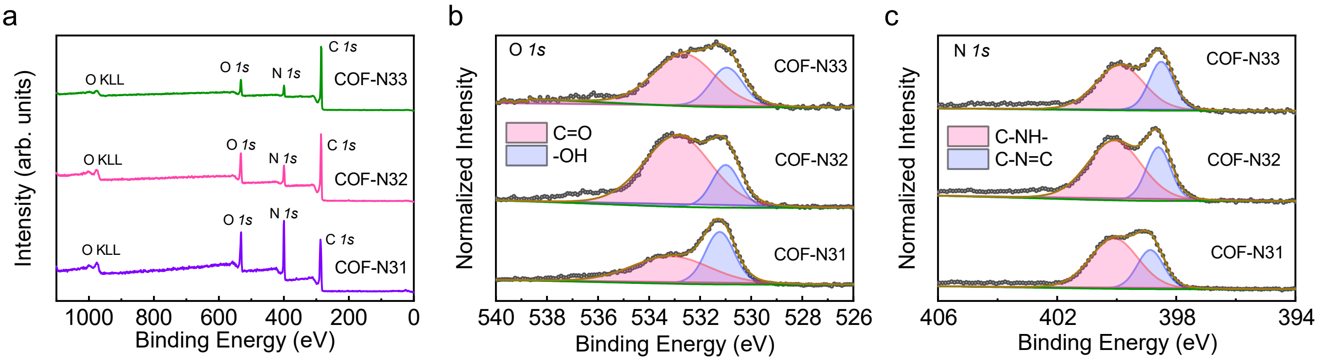

Figure S1. XPS spectra of three COFs. (a) Survey scan XPS profile, high-resolution XPS spectra of (b) O *1s* and (c) N *1s* of COFs. The O *1s* peaks can be divided into two peaks for absorbed –OH at 531.1 eV and C=O at 533.0 eV, respectively. The peaks at 398.6 eV and 400.0 eV in N *1s* spectra of COFs can be indexed to C–N=C and C–NH–, respectively.

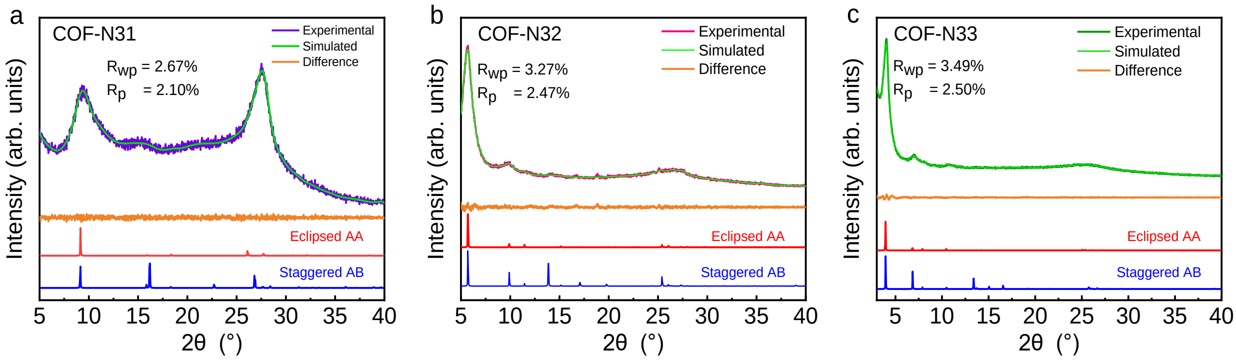

Figure S2. Experimental and simulated X-ray diffraction (XRD) patterns of (a) COF-N31, (b) COF-N32 and (c) COF-N33. Pawley refinements show the good agreement between experimental and simulated eclipsed XRD patterns (R_{wp} = 2.67%, R_p = 2.10% for COF-N31, R_{wp} = 3.27%, R_p = 2.47% for COF-N32, R_{wp} = 3.49%, R_p = 2.50% for COF-N33).

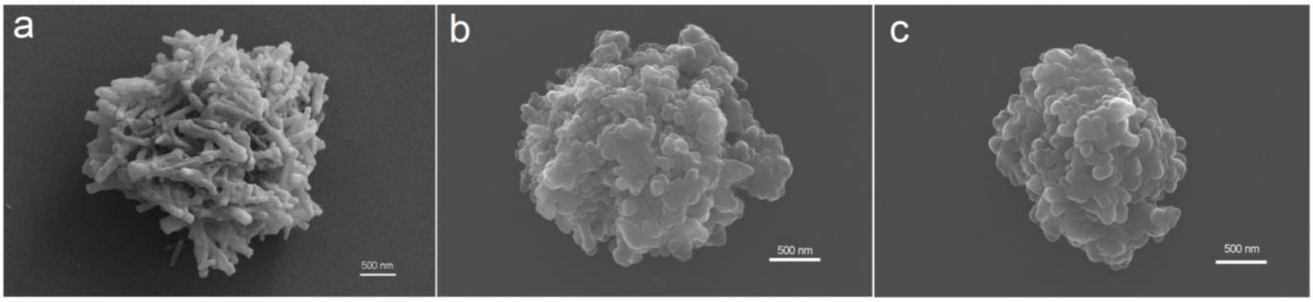

Figure S3. SEM images of (a) COF-N31, (b) COF-N32 and (c) COF-N33. SEM images show the spherical structure of three COFs with a diameter of 2–3 μm, which is assembled by numerous nanorods.

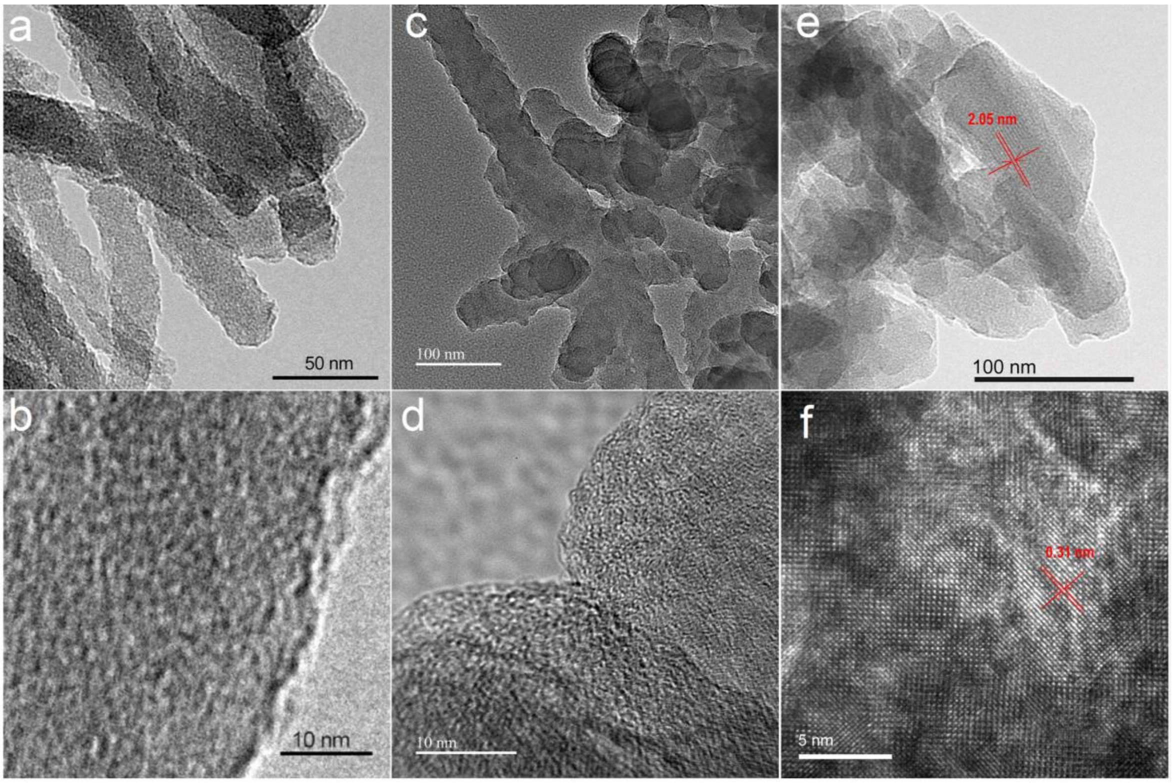

Figure S4. TEM images of (a, b) COF-N31, (c, d) COF-N32 and (e, f) COF-N33. The diffraction fringes can not be observed in COF-N31 and COF-N32 due to the relatively low crystallinity of COFs compared with inorganic semiconductors[S2, S4-5] . In COF-N33 with high crystallinity, the diffraction fringes of (100) and (002) planes can be found in **Figure S4e** and **S4f**, respectively.

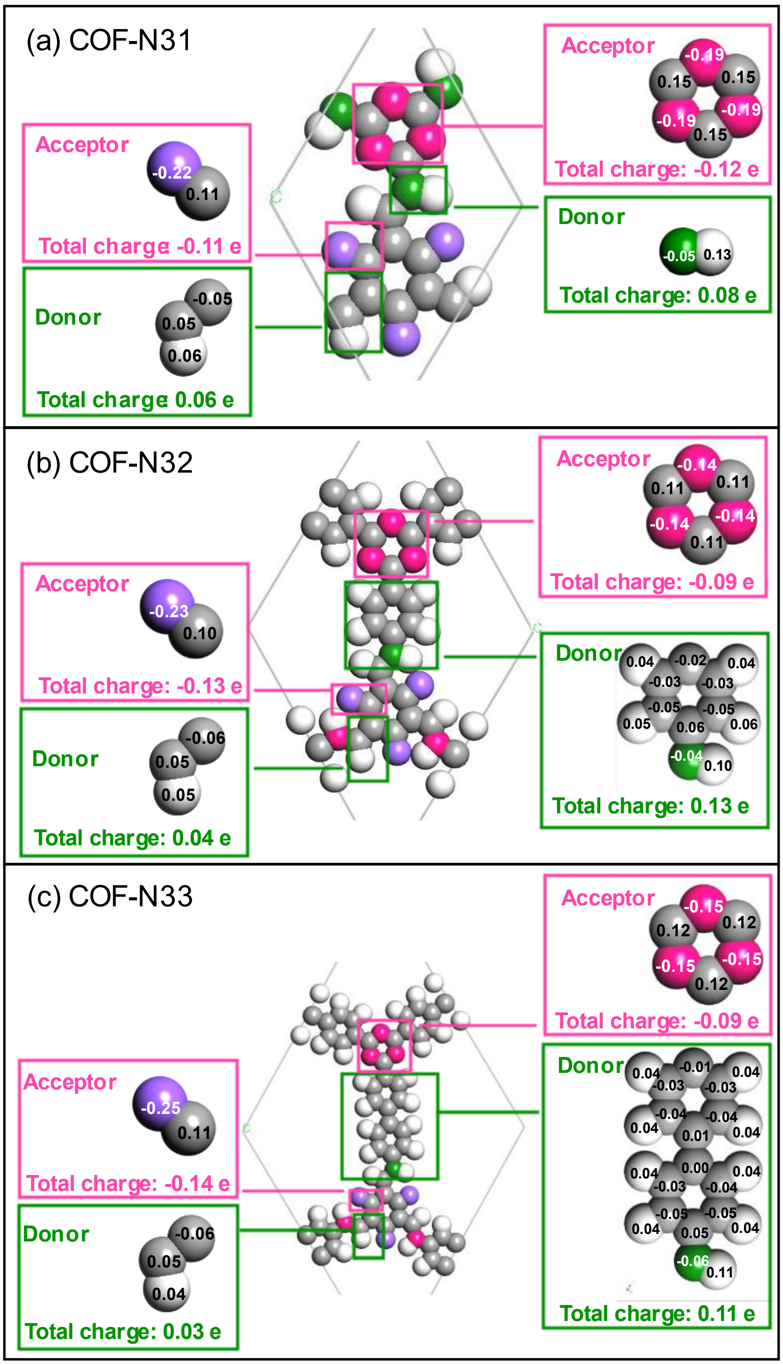

Figure S5. Charge contribution in (a) COF-N31, (b) COF-N32 and (c) COF-N33 structures.

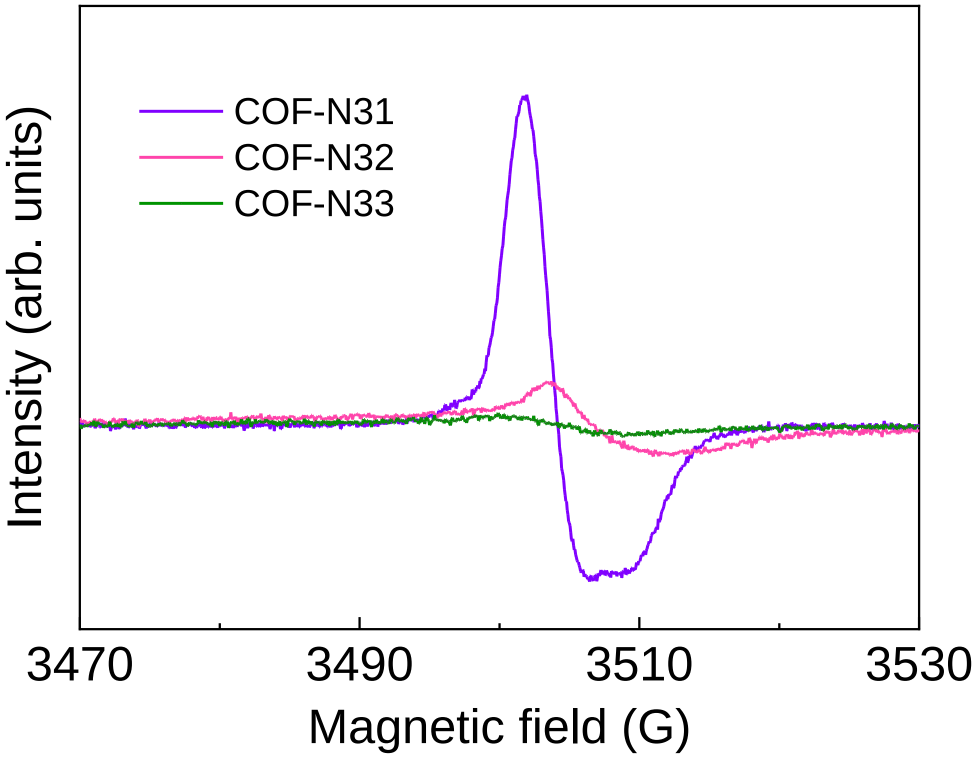

Figure S6. Solid-state electron spin resonance spectra of three COFs under dark and ambient conditions.

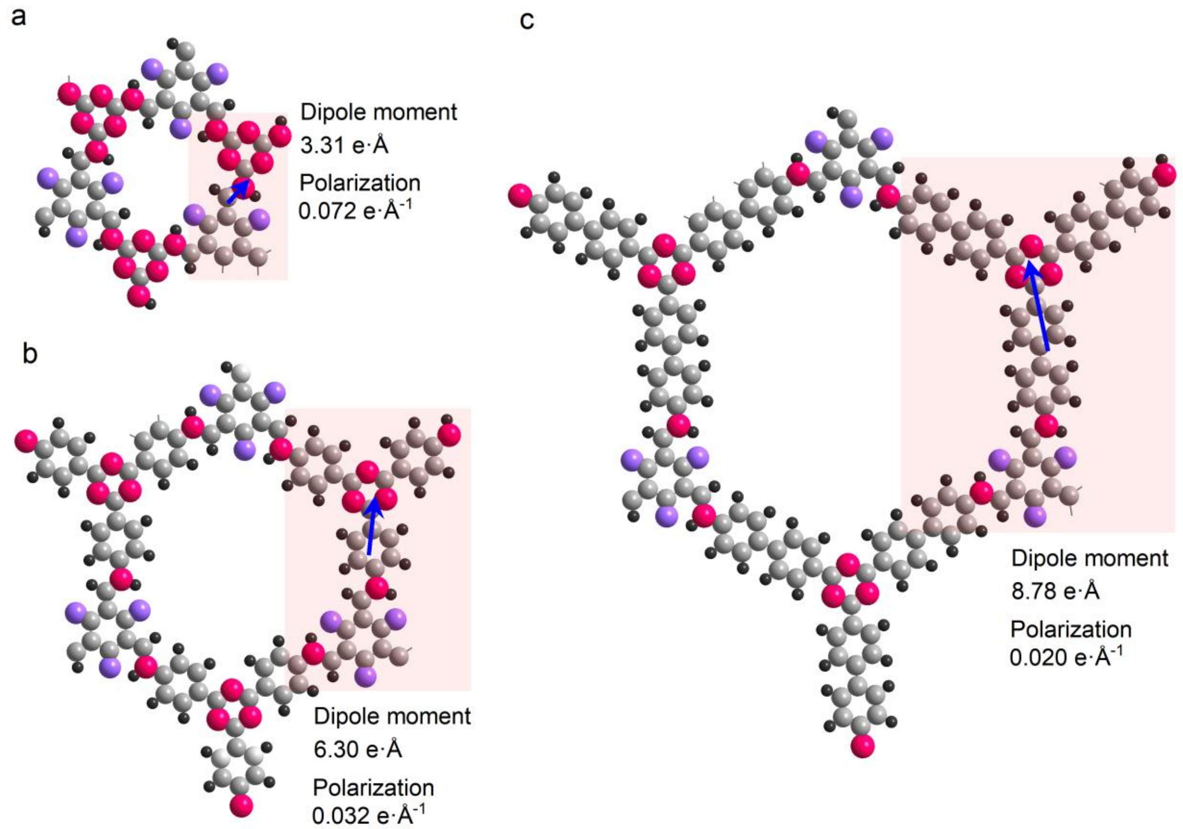

Figure S7. Dipole moment of the fragments in (a) COF-N31, (b) COF-N32 and (c) COF-N33.

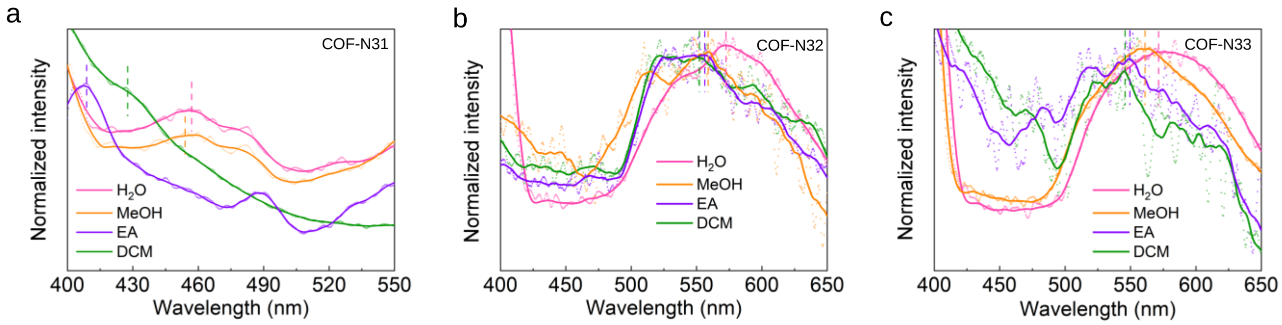

Figure S8. Solvatochromic behaviors of (a) COF-N31, (b) COF-N32 and (c) COF-N33 (MeOH-methanol, EA-ethyl acetate, DCM-dichloromethane).

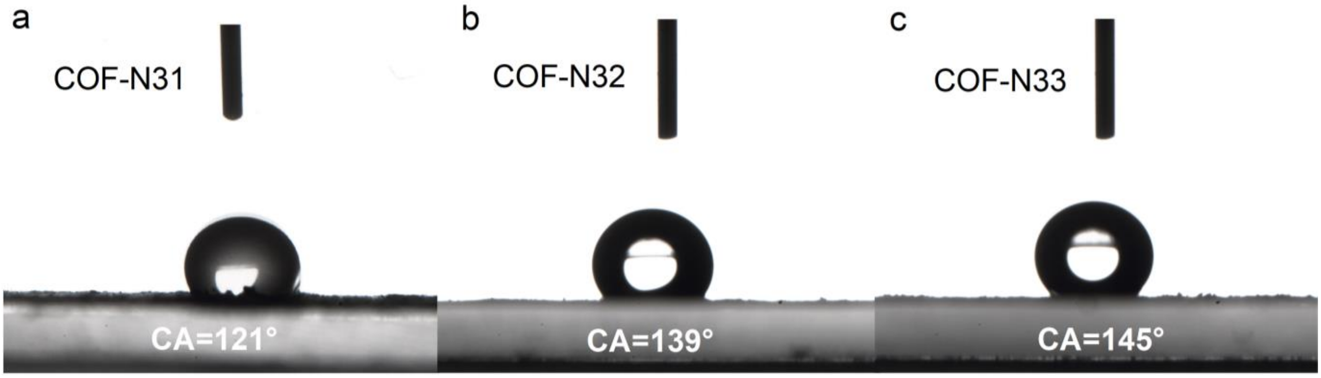

Figure S9. Water contact angles (CA) of water droplet on the pressed pellet of (a) COF-N31, (b) COF-N32 and (c) COF-N33.

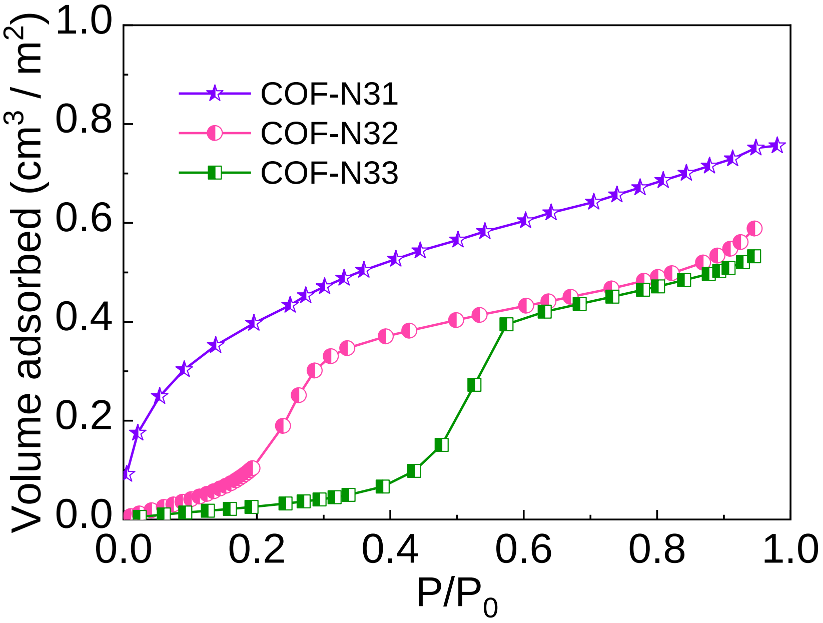

Figure S10. The water adsorption isotherms of three COFs, which is normalized by BET surface area.

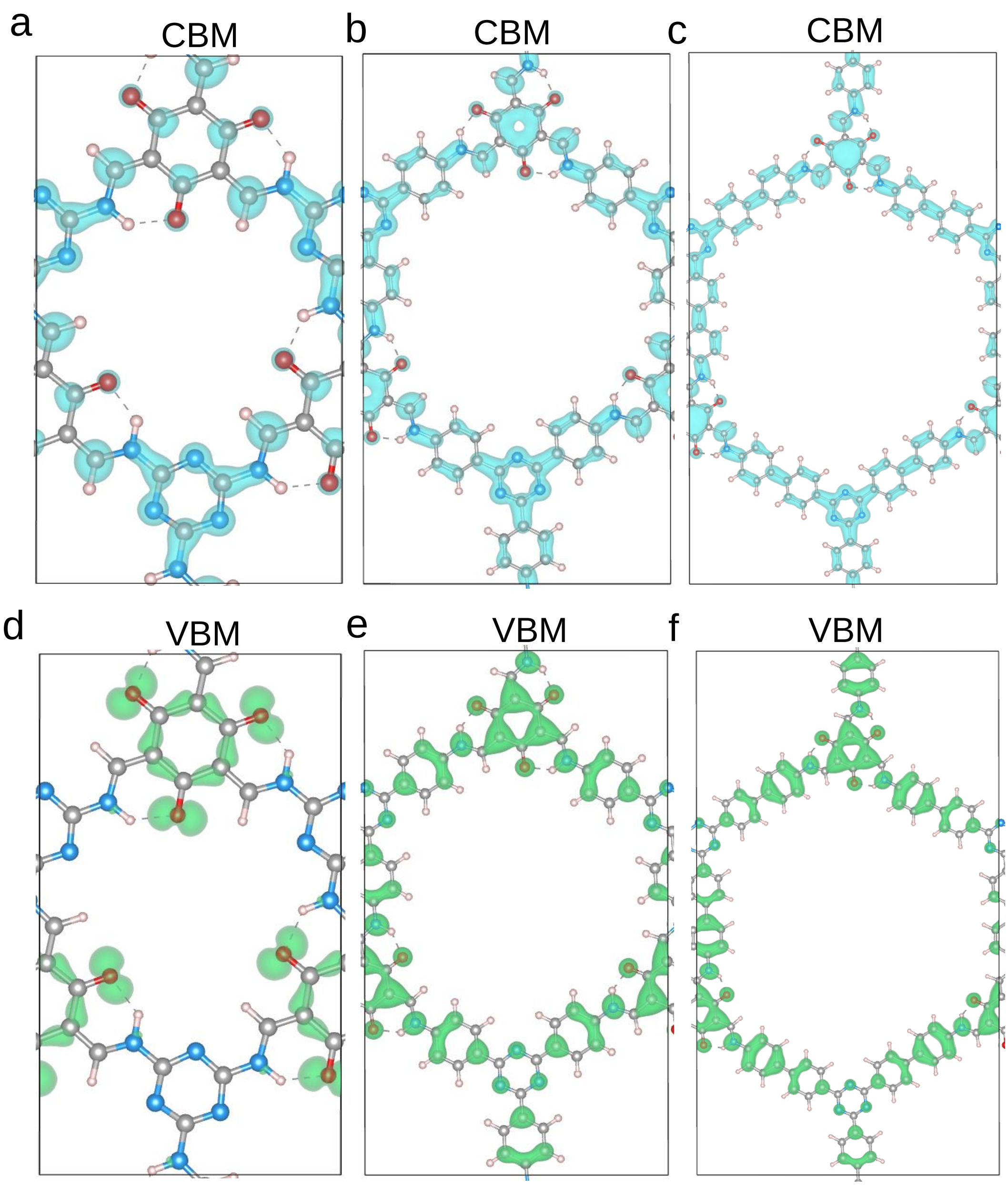

248

249 **Figure S11.** HOMO (up), LUMO (down) orbit distribution of (a,d) COF-N31, (b,e) COF-N32 and (c,f) COF-N33.
250

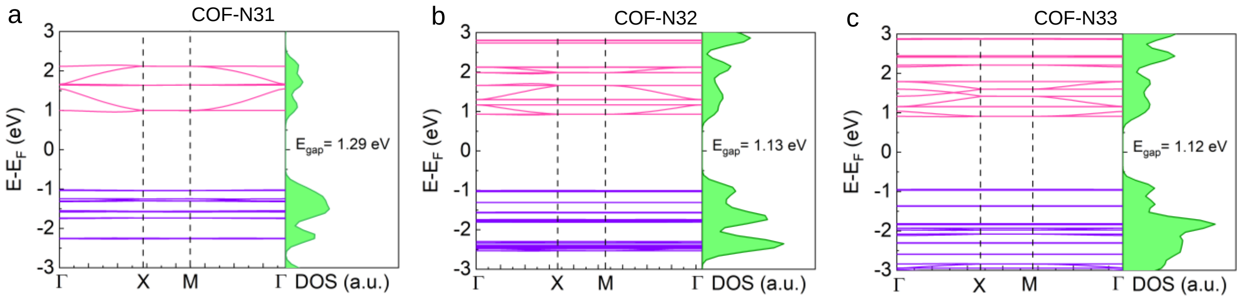

Figure S12. Calculated band structure and density of state (DOS) of (a) COF-N31, (b) COF-N32 and (c) COF-N33. Three COFs exhibit a direct bandgap at the Γ-point of the Brilloin zone. Note that slight degeneracy of conduction band edges of COF-N31 can be observed along Γ-X direction of the Brillouin zone while no degeneracy of the conduction band edges in COF-N32 and COF-N33. The observation can be attributed to the strong intramolecular polarity of COF-N31. Meanwhile, the calculated band gaps of three COFs follow the order of COF-N31 (1.29 eV) > COF-N32 (1.13 eV) > COF-N33 (1.12 eV), which is consistent with the order of band gaps calculated in Tauc plot.

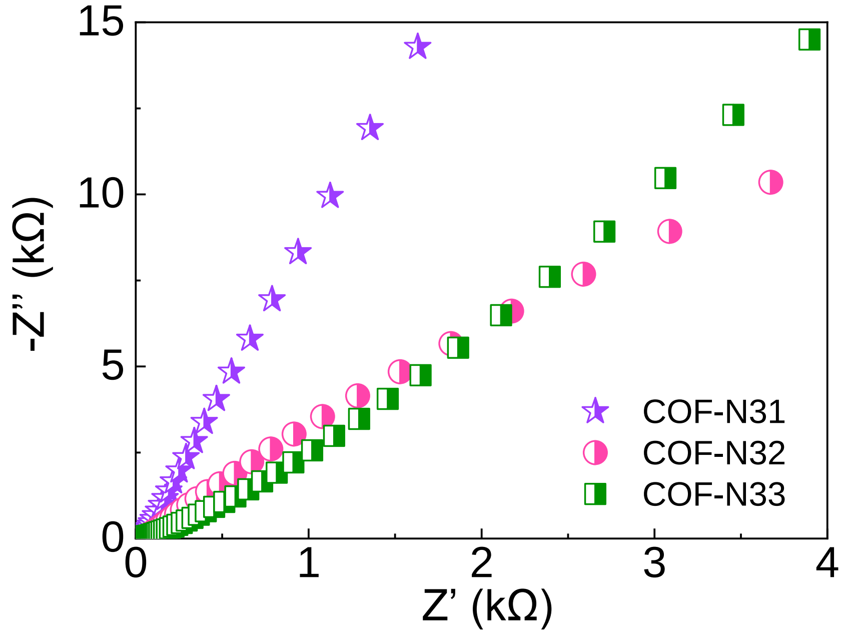

Figure S13. Electric impedance spectra of COF-N31, COF-N32 and COF-N33.

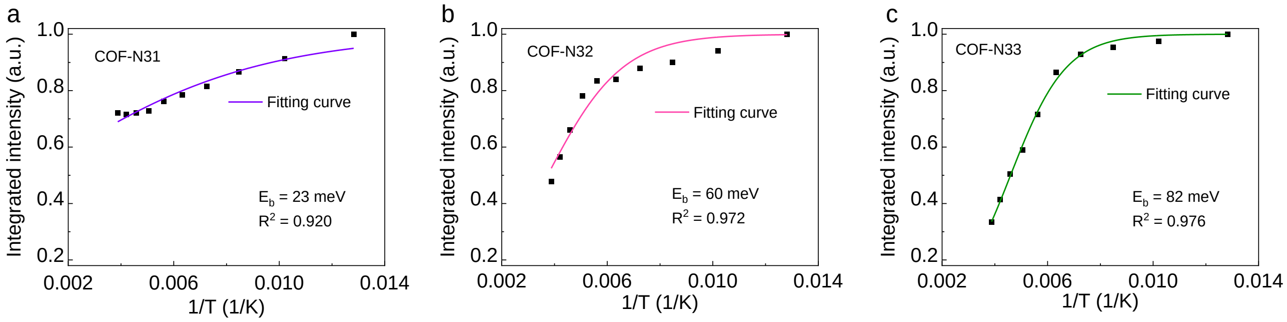

Figure S14. Integrated PL intensities as a function of temperature of (a) COF-N31, (b) COF-N32 and (c) COF-N33.

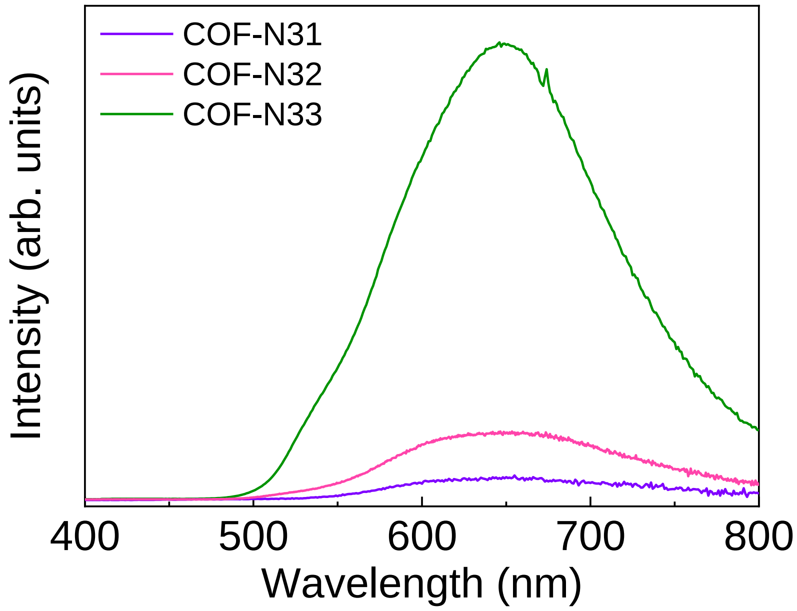

Figure S15. Steady-state PL spectra of three COFs excited at 365 nm.

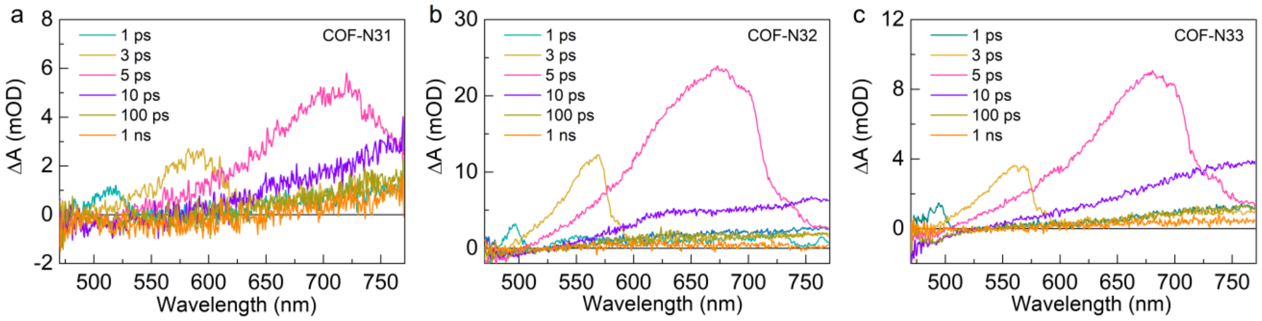

Figure S16. Transient adsorption spectra of (a) COF-N31, (b) COF-N32 and (c) COF-N33 registered at different probe delays (pump at 360 nm).

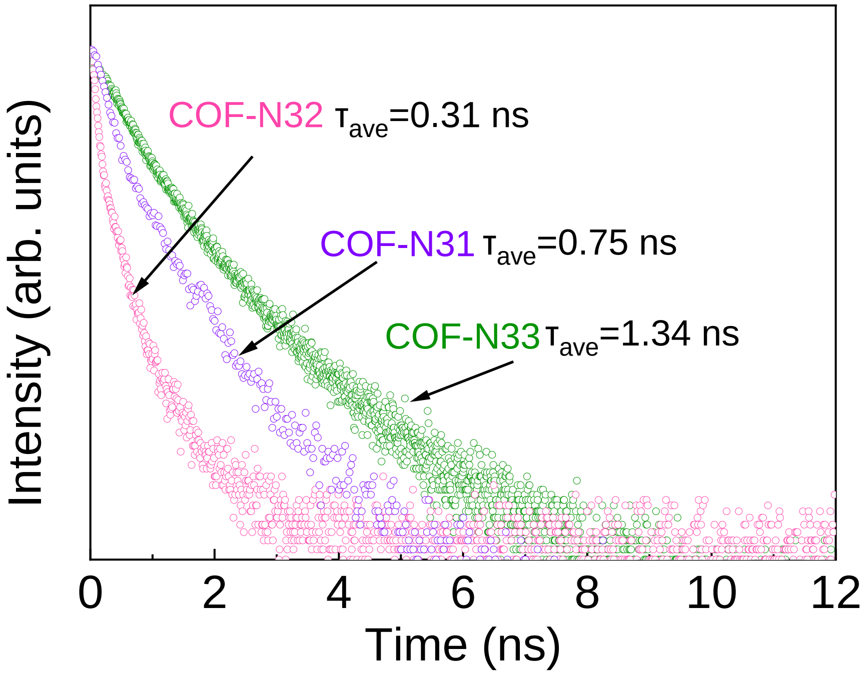

Figure S17. PL decay curves of three COFs.

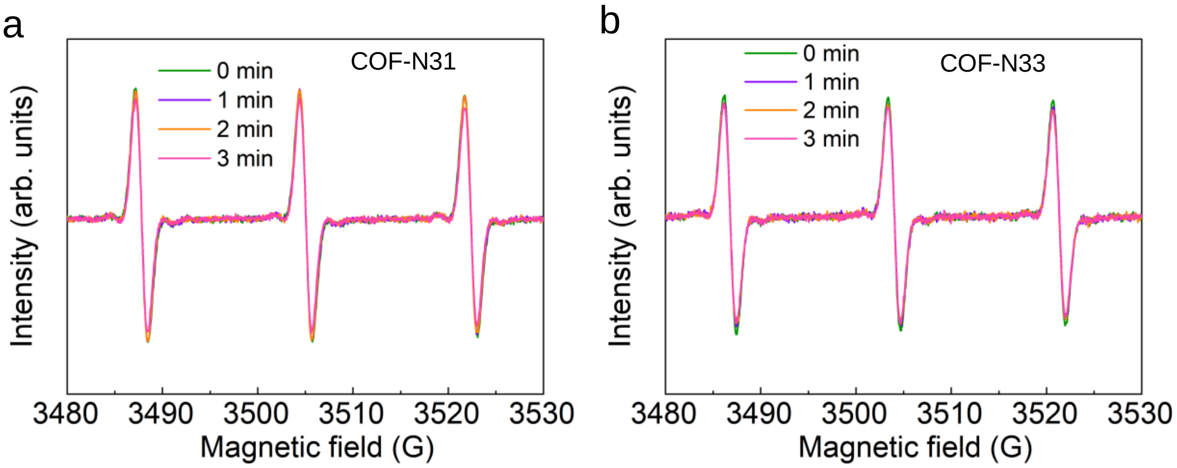

Figure S18. ESR spectra of TEMPO for the detection of photo-generated free charges in (a) COF-N31 and (b) COF-N33.

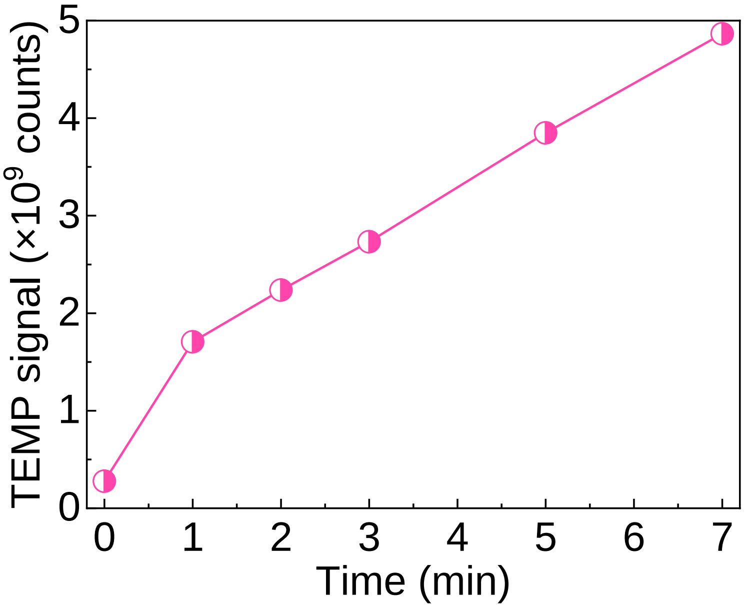

Figure S19. Generation of TEMP from TEMPO by the photo-induced electrons in COF-N32 under visible light irradiation.

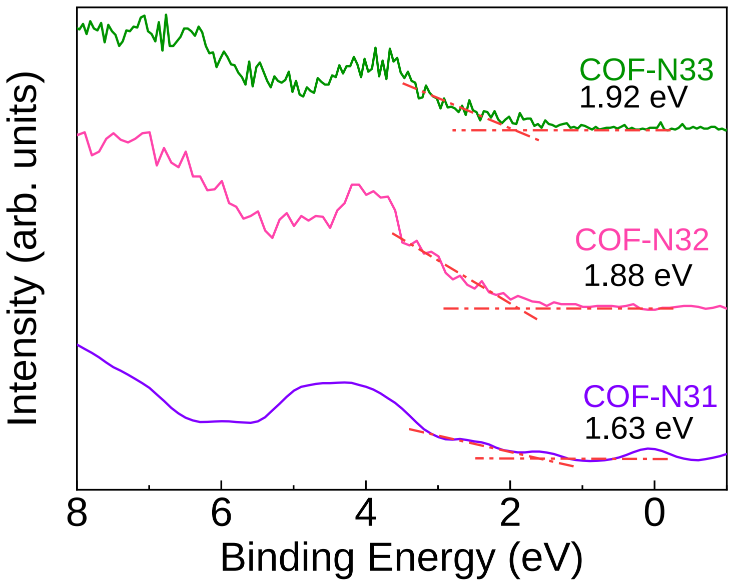

Figure S20. Valence band XPS spectra of three COFs. The equilibration of the Fermi level of the instrument was conducted at 4.5 eV by using Au metal basis as the reference. The potentials calculated by binding energy in VB-XPS spectra equals to the VB potential (vs. NHE). Thus, the VB potentials of COF-N31, COF-N32 and COF-N33 are determined to be 1.63 V vs. NHE, 1.88 V vs. NHE and 1.92 V vs. NHE, respectively.

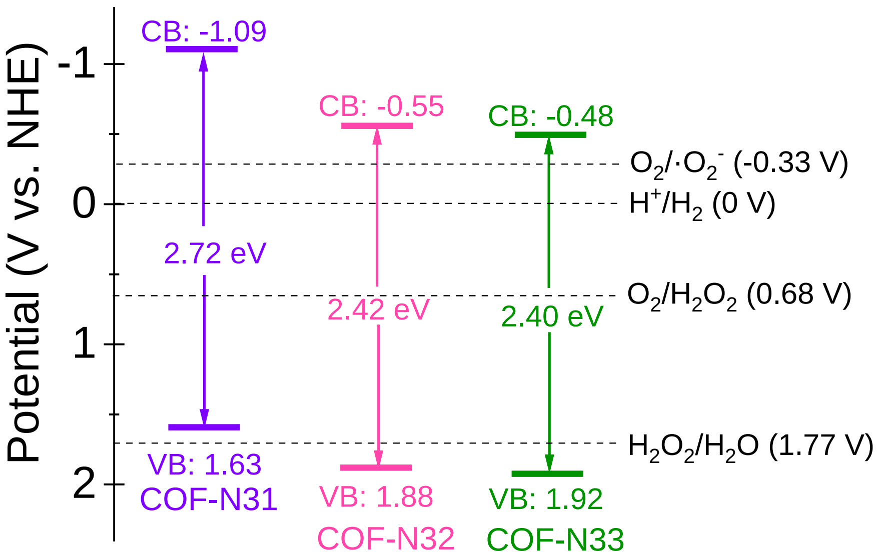

Figure S21. Schematic illustration of energy bands of three COFs. According to the VB-XPS results and the band gaps calculated in Tauc plot (Figure 2a), the energy band position of COFs can be determined, which confirms that the oxygen reduction reaction by COFs are thermodynamically feasible. The two-electron water oxidation reaction (WOR) directly to H_2O_2 (1.77 V vs. NHE) is also thermodynamically possible by COF-N32 (1.88 V vs. NHE) and COF-N33 (1.92 V vs. NHE), but not by COF-N31 (1.63 V vs. NHE). Instead, h^+ generated by COF-N31 can attack COF itself in pure water without scavengers.

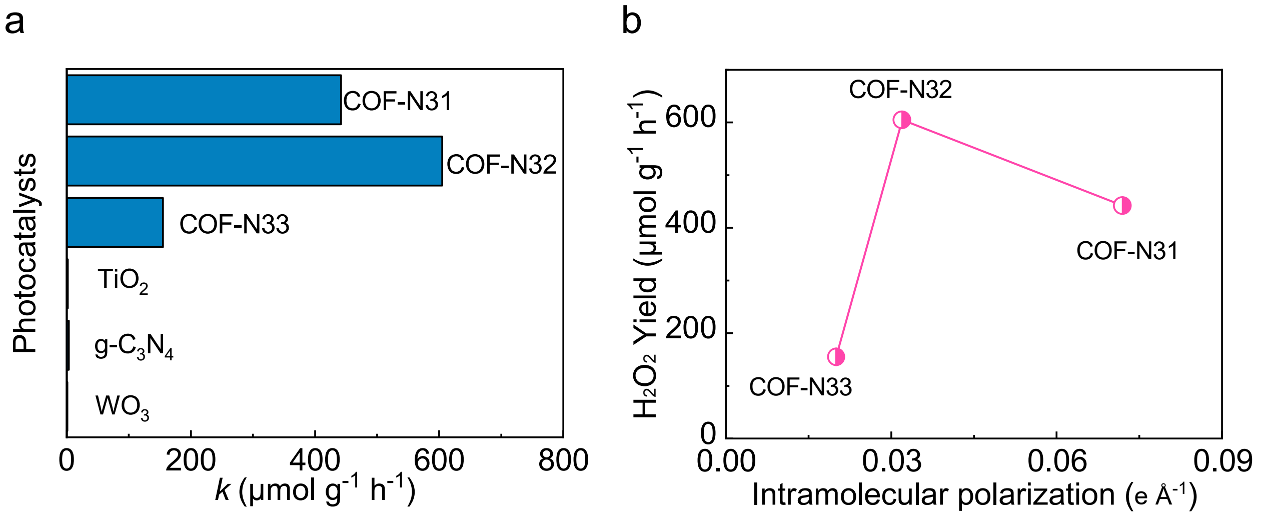

Figure S22. The relationship between H_2O_2 photosynthesis kinetics and intramolecular polarization for three COFs. (a) H_2O_2 photosynthesis kinetics by different photocatalysts under visible light irradiation; (b) H_2O_2 photosynthesis kinetics as a function of intramolecular polarization for three COFs.

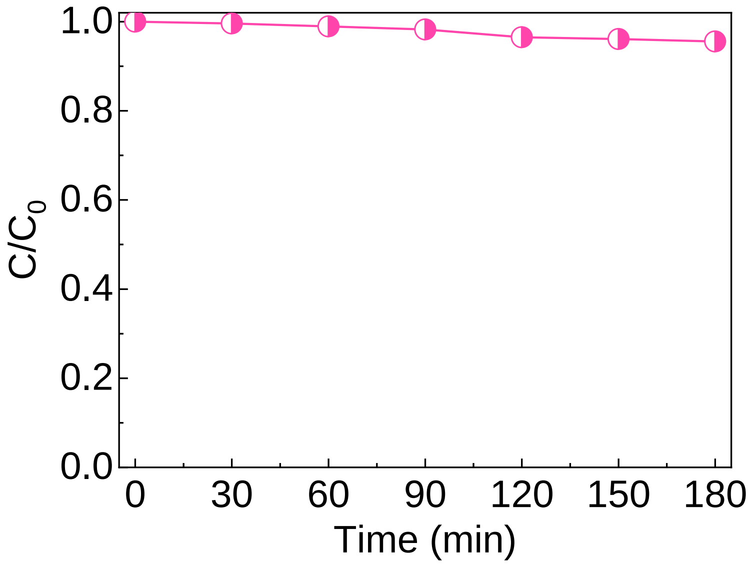

Figure S23. Degradation of 1 mM H_2O_2 by COF-N32 under N_2 atmosphere. Conditions: λ>420 nm (298K; xenon lamp, light intensity:100 mW·cm^{-2}), H_2O_2 solution (1 mM, 50 mL), photocatalyst (25 mg).

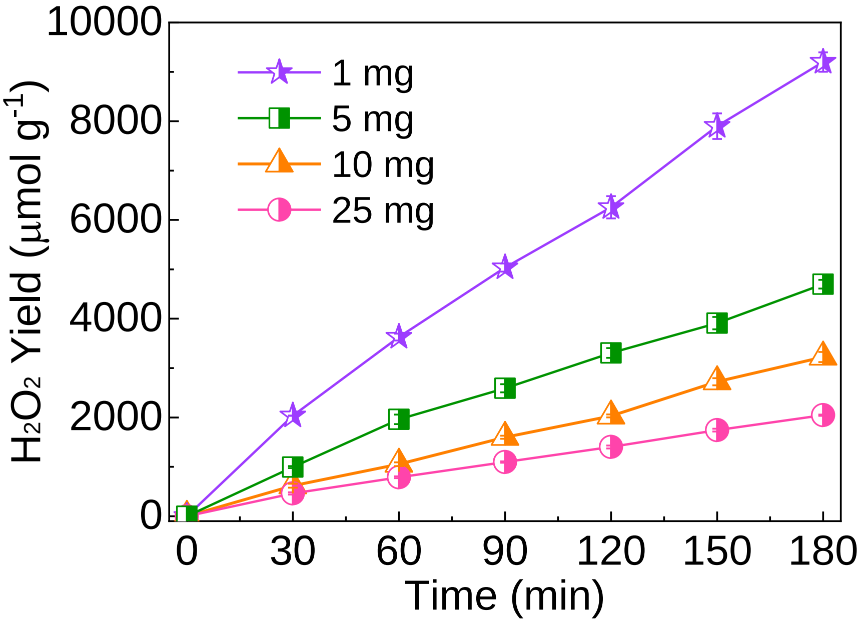

Figure S24. H_2O_2 yield by COF-N32 with different dosage in 50 mL O_2-saturated ultrapure water. Conditions: λ>420 nm (298K; xenon lamp, light intensity:100 mW·cm^{-2}), ultrapure water (50 mL). COF-N32 can yield over 3168 µmol g^{-1} h^{-1}, 1612 µmol g^{-1} h^{-1} and 1068 µmol g^{-1} h^{-1} with the addition of 1, 5 and 10 mg COF-N32 in 50 mL ultrapure water, respectively, which are much higher than those of recently reported photocatalysts in pure water under the similar measurement conditions (**Table S3**). Error bars represent the average values (mean ± s.d., *n*=3).

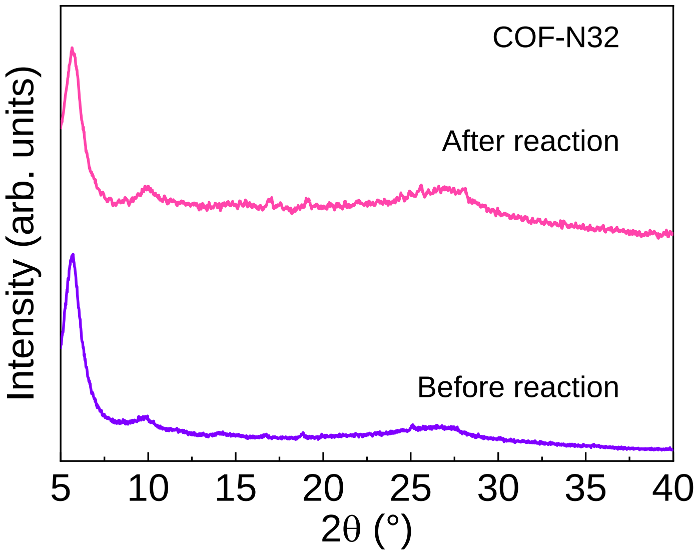

Figure S25. XRD patterns of COF-N32 before reaction and after reaction.

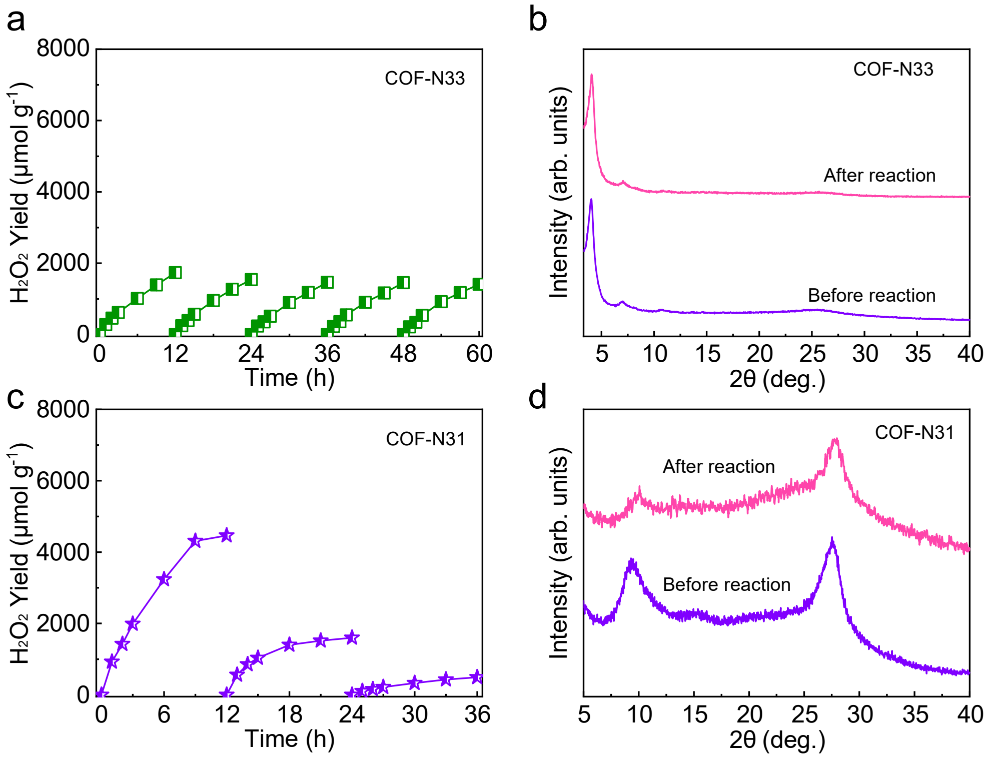

Figure S26. The reusability of (a) COF-N33 and (c) COF-N31 for H_2O_2 photosynthesis. XRD patterns of (b) COF-N33 and (d) COF-N31 before and after reaction. Conditions: λ>420 nm (298K; xenon lamp, light intensity:100 mW·cm^{-2}), ultrapure water (50 mL), photocatalyst (25 mg).

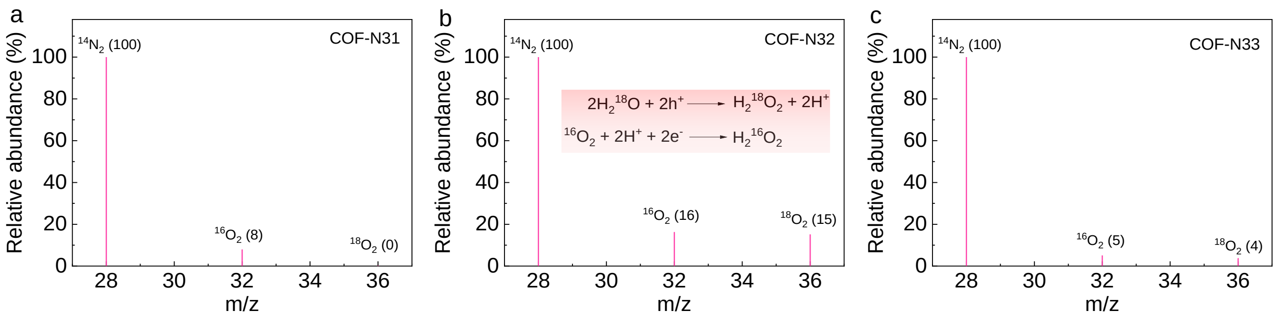

Figure S27. Isotopic experiment by using $H_2{}^{18}O$ as water source during H_2O_2 photosynthesis by (a) COF-N31, (b) COF-N32 and (c) COF-N33.

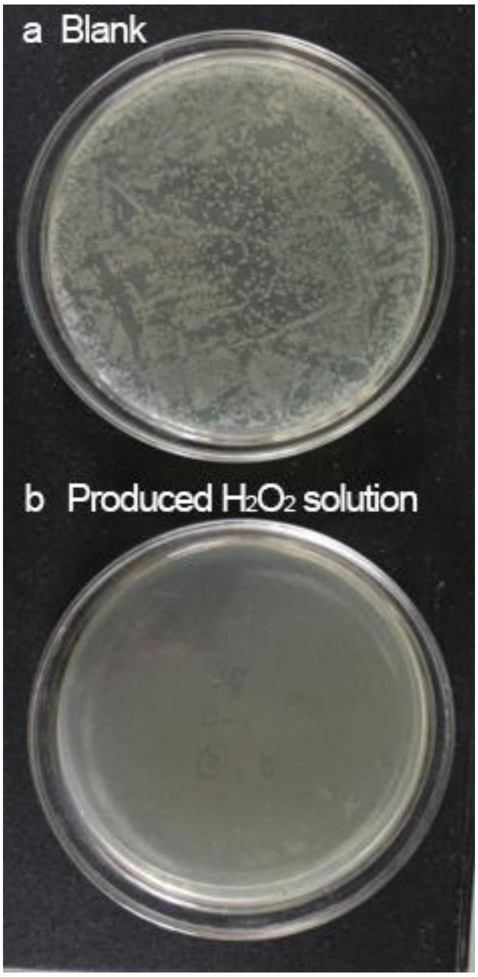

Figure S28. Photographs of culture media for the disinfection of antibiotic-resistant bacteria (a) without and (b) with the mixture of H_2O_2 solution produced by COF-N32.

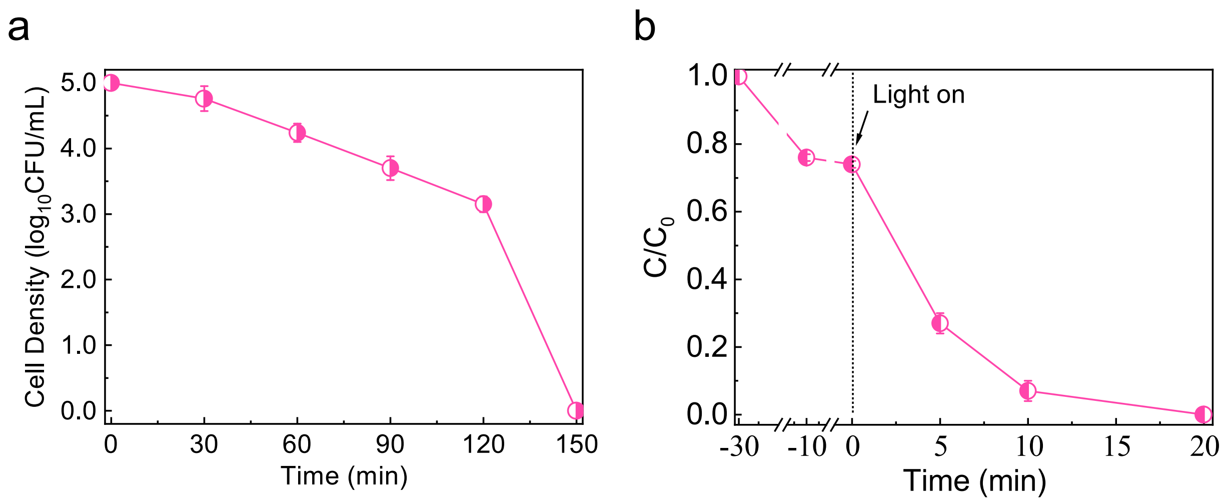

Figure S29. The in-situ photocatalytic bacterial disinfection and diclofenac degradation by COF-N32 under visible light irradiation. (a) In-situ photocatalytic disinfection of antibiotic-resistant bacteria by COF-N32 under visible light irradiation. (b) In-situ photocatalytic degradation of diclofenac by COF-N32 under visible light irradiation. Conditions: λ>420 nm (298K; xenon lamp, light intensity:100 mW·cm^{-2}), 50 mL of reaction suspension containing 5 mg of COF-N32. Error bars in (a) and (b) represent the average values (mean ± s.d., n=3).

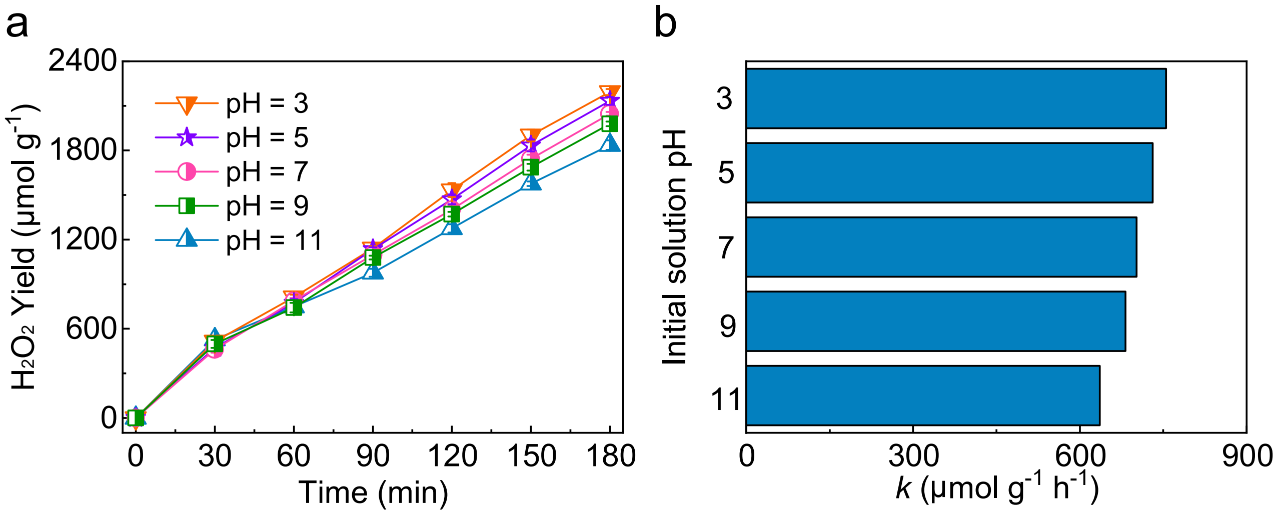

Figure S30. Effects of initial solution pH on (a) H_2O_2 yield and (b) corresponding formation kinetics by COF-N32 under visible light irradiation. Conditions: λ>420 nm (298K; xenon lamp, light intensity:100 mW·cm^{-2}), ultrapure water (50 mL), COFs (25 mg). Error bars in (a) represent the average values (mean ± s.d., *n*=3).

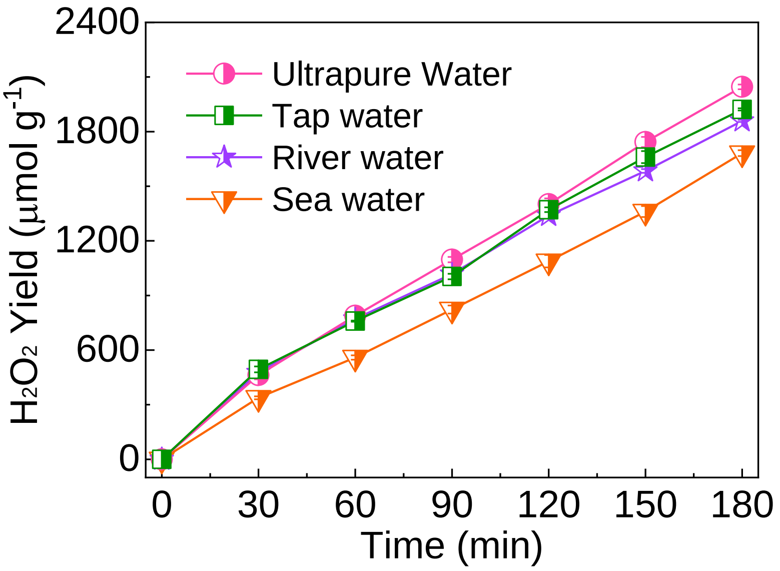

Figure S31. Photocatalytic H_2O_2 production by COF-N32 in ultrapure water, tap water, river water and sea water under visible light irradiation. Conditions: λ>420 nm (298K; xenon lamp, light intensity:100 mW·cm^{-2}), real water samples (50 mL), COFs (25 mg). Error bars represent the average values (mean ± s.d., n=3).

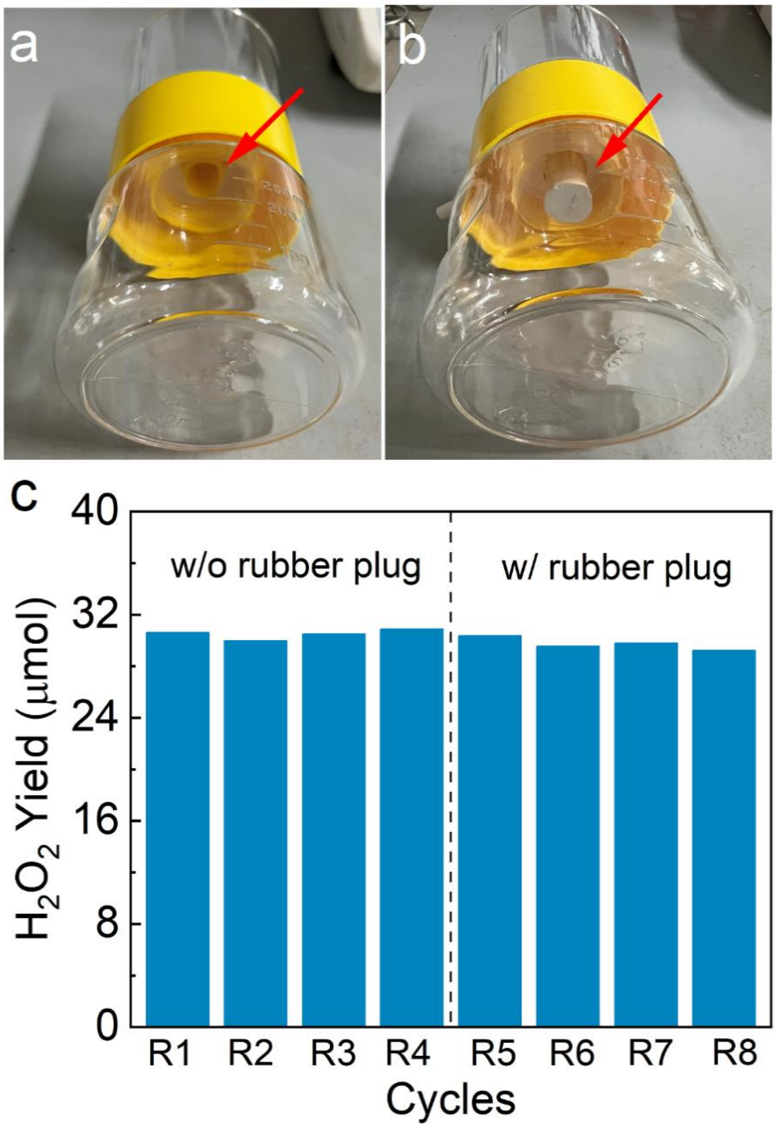

Figure S32. H_2O_2 photosynthesis by COF-N32 in a membrane reactor under visible light irradiation. Photographs of membrane filter reactor (a) without and (b) with rubber plug in the filter. (c) Photocatalytic production of H_2O_2 by COF-N32 in a membrane reactor without and with rubber plug. Without the rubber plug in the filter, the generated H_2O_2 can be gradually filtered through the 0.22 μm membrane under gravity within 2 h (**Figure S32a**). The overall H_2O_2 yield in filtrate remains to be ~30 μmol for four cycles, confirming the stability of COF-N32 under visible light irradiation. When the filter is blocked by rubber plug, the liquid can be kept in the upper reactor (**Figure S32b**). After the reaction, the generated H_2O_2 can be obtained via the vacuum filtration without the loss of COF-N32 for the successive cycle. The H_2O_2 yield by COF-N32 was also ~30 μmol in another four cycles. The above results show that COF-N32 in the membrane reactor can continuously produce H_2O_2 under visible light irradiation only with the addition of pure water.

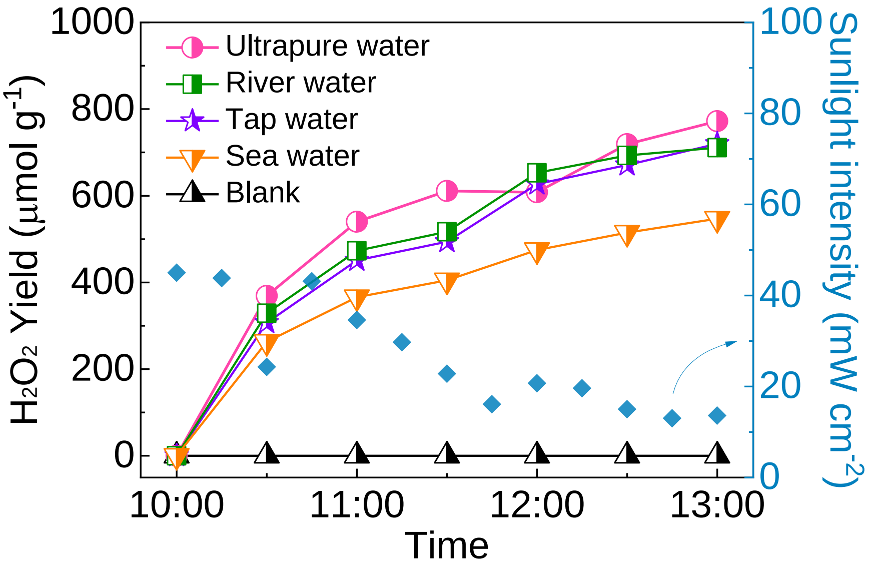

Figure S33. Photocatalytic production of H_2O_2 by COF-N32 in different real water samples under natural solar irradiation with the reaction temperature controlled at 25 °C on Feb. 17 (a cloudy day). The photocatalytic H_2O_2 production kinetics on Apr. 7 (a sunny day) in different water samples follow the order of ultrapure water (602 μmol g^{-1} h^{-1}) > river water (515 μmol g^{-1} h^{-1}) > tap water (475 μmol g^{-1} h^{-1}) > sea water (396 μmol g^{-1} h^{-1}) (**Figure 4a**). The slightly decreased yields in real water samples can be attributed to the slight inhibition on H_2O_2 yield by NOM and ions. Meanwhile, the H_2O_2 yield on Apr. 7 is higher than that on Feb. 17, which can be attributed to the strong light irradiation on Apr. 7 relative to Feb. 17 (**Figures 4a and S33**). However, great amount of H_2O_2 (19.3 μmol) can still be produced within 3 h on Feb. 17. The results show that COF-N32 can efficiently photosynthesize H_2O_2 in real water samples under natural sunlight irradiation.

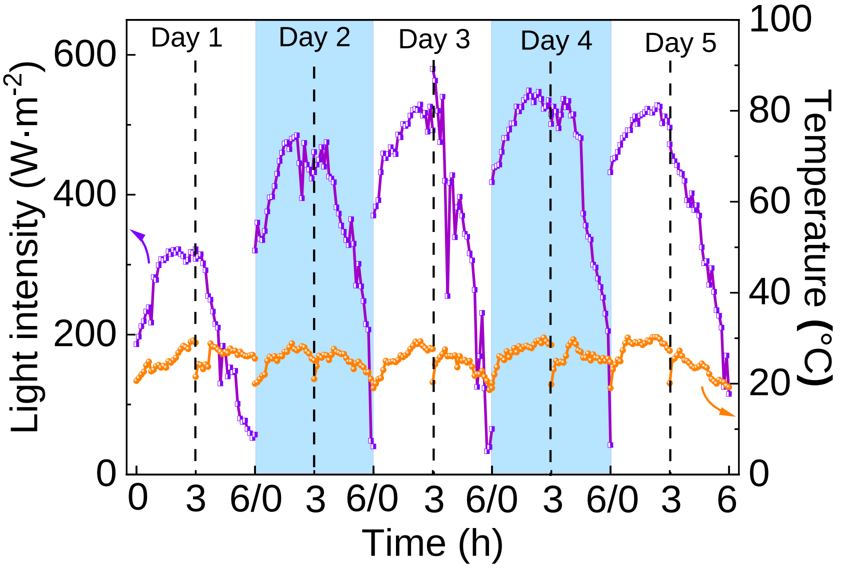

Figure S34. Light intensities of natural solar irradiation during the H_2O_2 photosynthesis by COF-N32 in membrane reactors under natural sunlight irradiation.

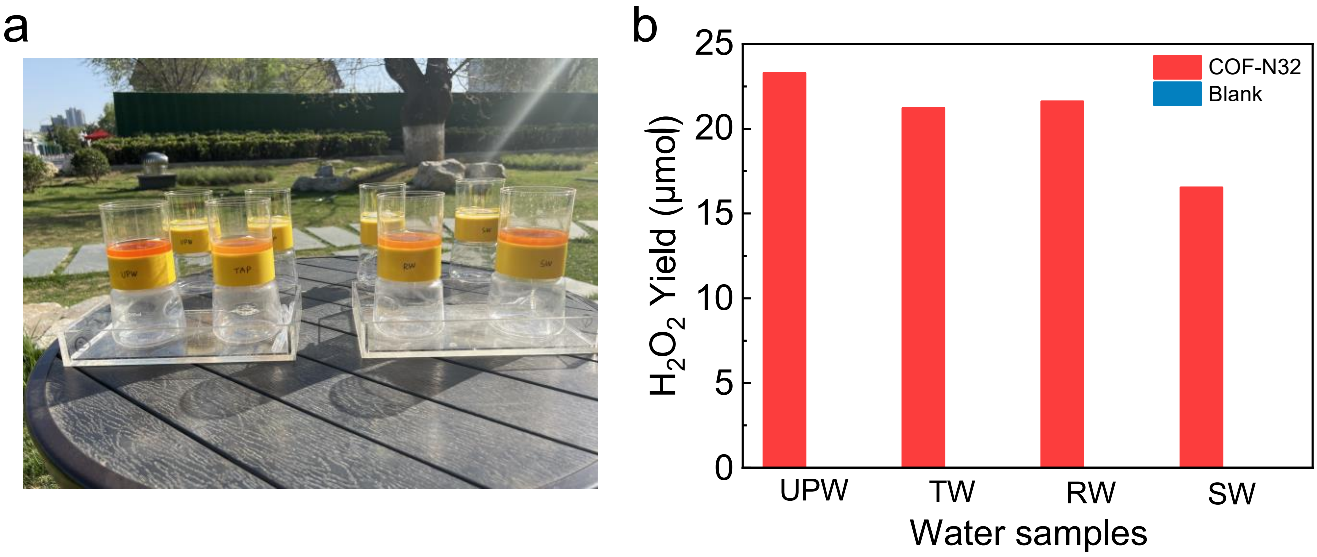

Figure S35. H_2O_2 yield in different types of water samples by COF-N32 in membrane reactor under natural solar irradiation. (a) Photographs of membrane filter reactors. (b) H_2O_2 yield by COF-N32 in ultrapure water (UPW), tap water (TW), river water (RW) and sea water (SW). Under natural sunlight irradiation (**Figure S34**), ~20 μmol of H_2O_2 can be efficiently generated in four real water samples while negligible amount of H_2O_2 can be generated without the addition of COF-N32 into the membrane filter reactors.

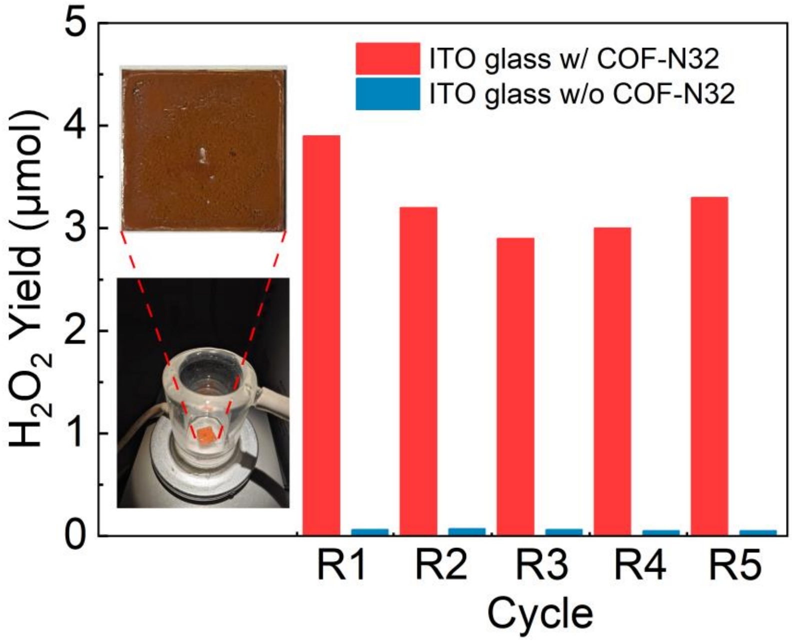

Figure S36. Immobilization of COF-N32 powders (5 mg) onto ITO glass substrate (2 cm×2 cm) for H_2O_2 production under visible light irradiation. Conditions: λ>420 nm (298K; xenon lamp, light intensity:100 mW·cm^{-2}), ultrapure water (10 mL), reaction time (2 h).

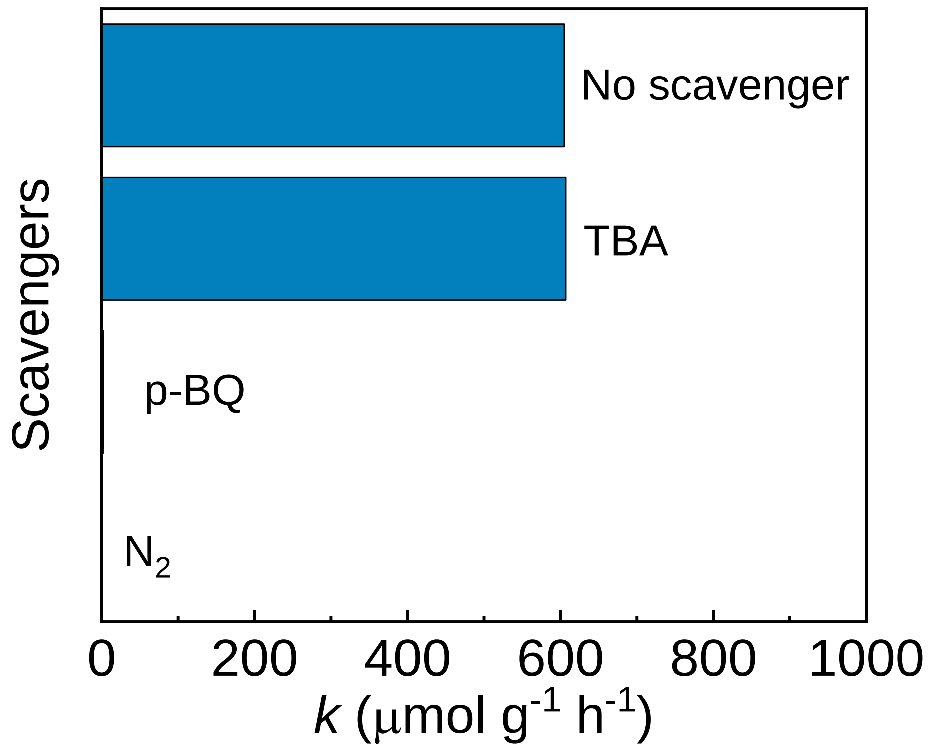

Figure S37. Effects of scavengers on the H_2O_2 photosynthesis kinetics by COF-N32 under visible light irradiation.

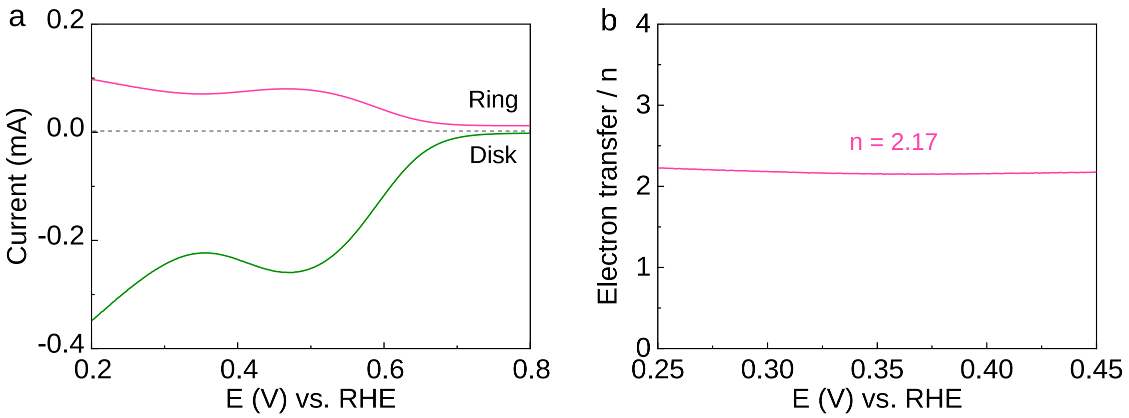

Figure S38. Rotating ring-disk electrode (RRDE) analysis of COF-N32. (a) RRDE curves over COF-N32-coated electrodes measured at 1600 rpm in O_2-saturated electrolyte using the ring current (top) and the disk current (bottom). (b) The average number of the transferred electrons (n) at different potentials calculated from RRDE data.

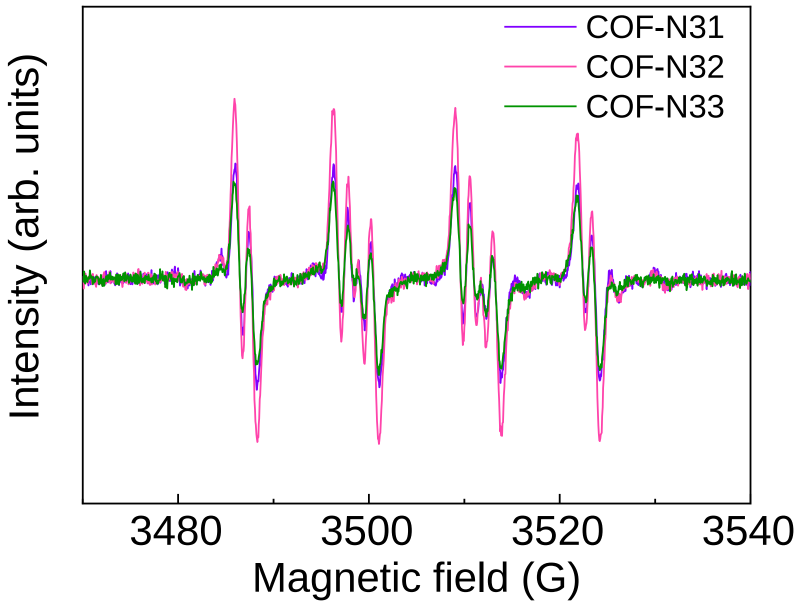

Figure S39. In-situ ESR spectra of DMPO-$\cdot O_2^-$ for COF-N31, COF-N32 and COF-N33 under 5 min of visible light irradiation.

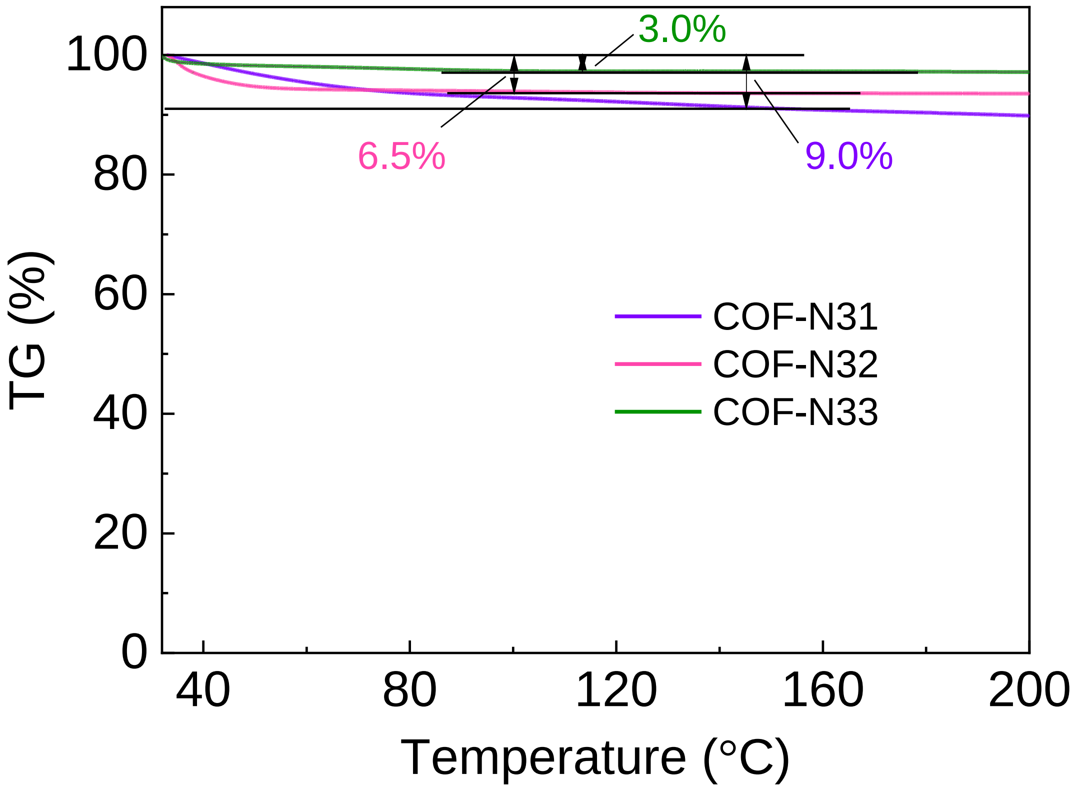

Figure S40. Thermogravimetric curves of COF-N31, COF-N32 and COF-N33. Less than 10% weight loss of COFs was observed under 200 °C, which can be attributed to the loss of adsorbed H_2O on the surface of COFs.

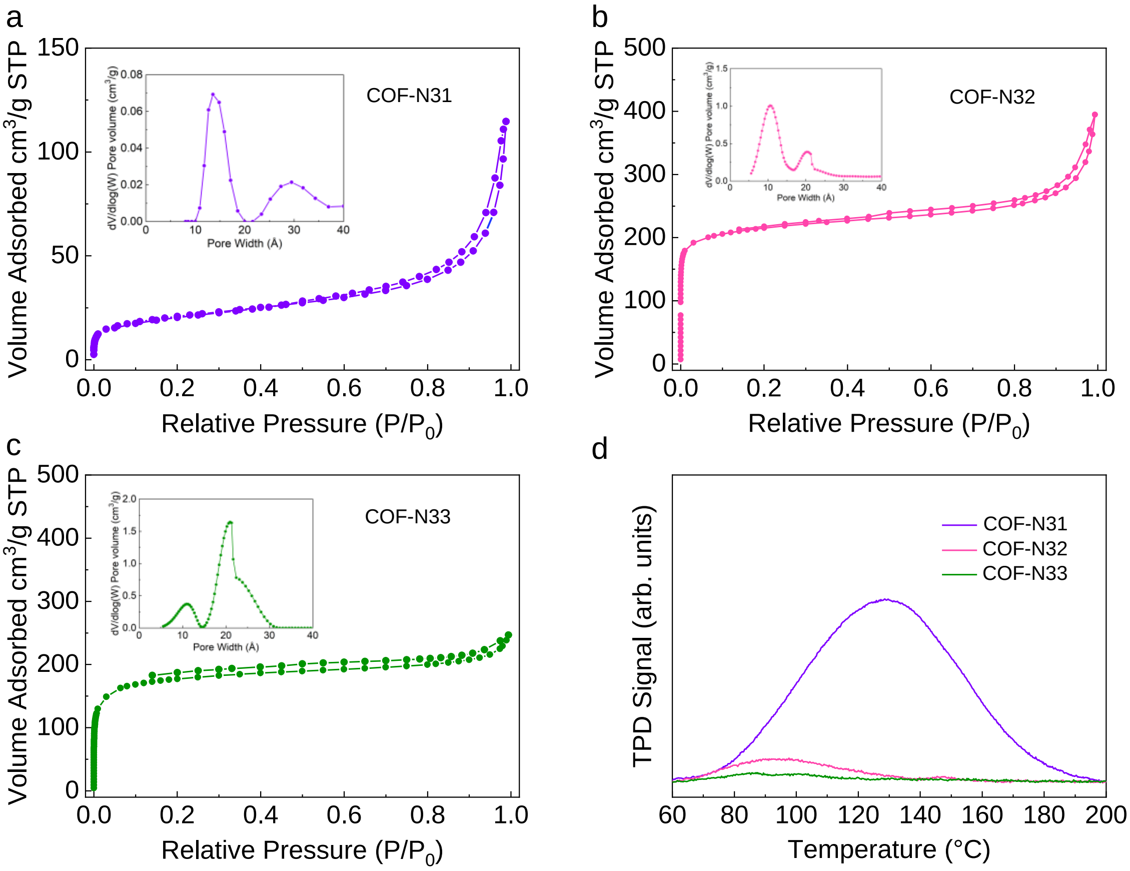

Figure S41. N_2 adsorption-desorption isotherms of (a) COF-N31, (b) COF-N32 and (c) COF-N33 and the corresponding pore size distribution (insets). (d) O_2-TPD curves of three COFs. The BET surface area of COF-N31, COF-N32 and COF-N33 is determined to be 75 m^2/g, 823 m^2/g and 677 m^2/g, respectively. The three COFs shows narrow pore size of 1-2 nm, indicating their micropore structure. The intensities of O_2-TPD signals follow the order of COF-N31 > COF-N32 > COF-N33. It should be noted that the COFs are thermally stable at the tested temperature (< 200°C, **Figure S40**), suggesting the signals in O_2-TPD are not resulted from the decomposition of COFs. Therefore, the O_2 adsorption is mainly relevant to the structure of COFs instead of surface area. Besides, COF-N31 with more O_2 adsorption exhibits relatively low production capability of $\cdot O_2^-$ and H_2O_2 compared with that of COF-N32, further implying that the electron transfer efficiency in oxygen reduction is more important than surficial properties during H_2O_2 production process.

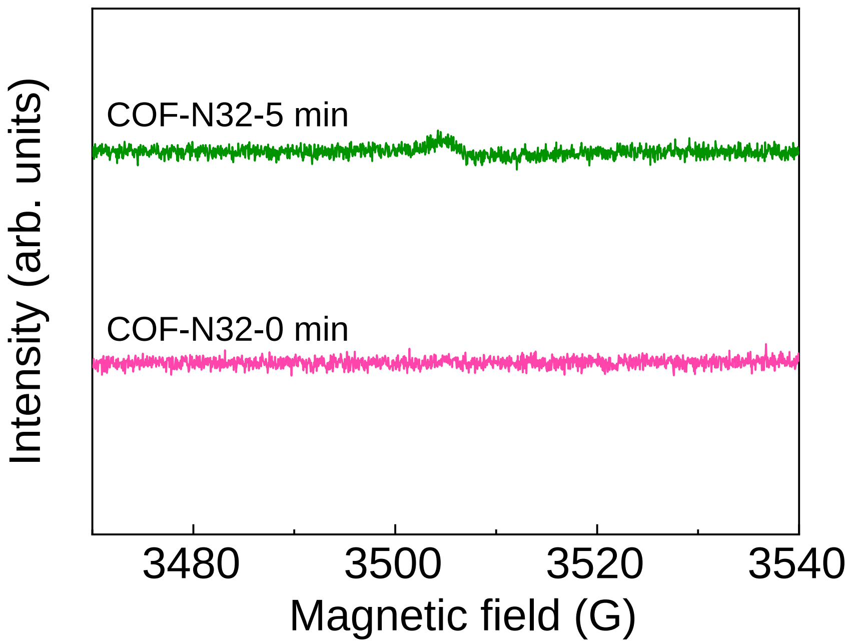

Figure S42. ESR spectra for DMPO-·OH in water before and after visible light irradiation. The typical quartet peaks for DMPO-·OH can hardly be observed in the ESR spectra of COF-N32 before and after light illumination, suggesting the absence of ·OH.

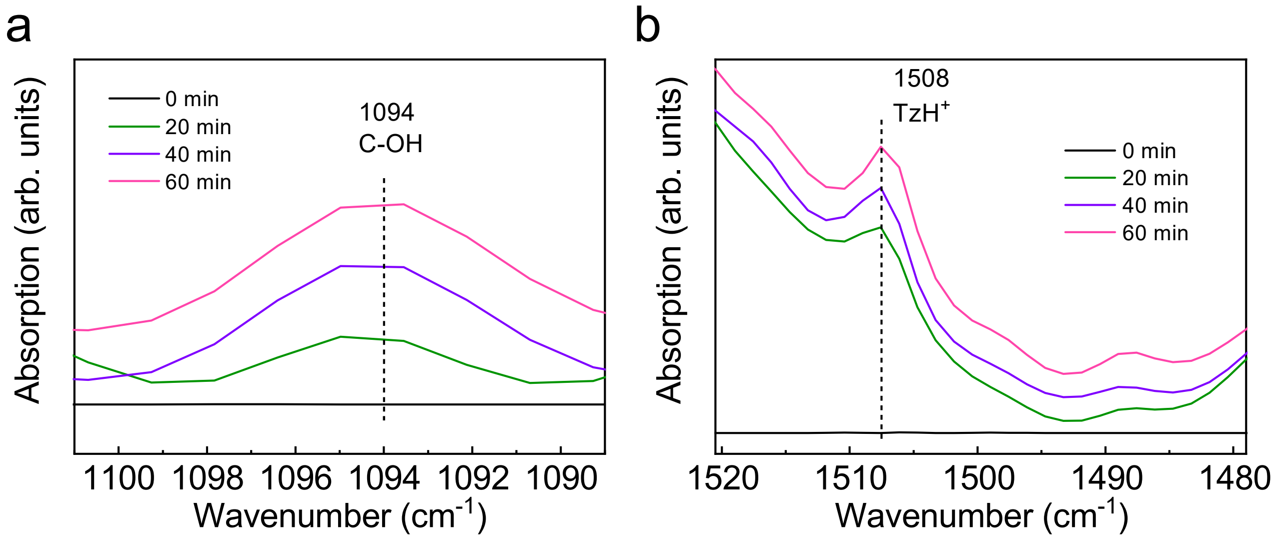

Figure S43. Time-course in-situ FTIR spectra of COF-N32 with H_2O under dark condition at (a) 1094 cm^{-1} and (b) 1508 cm^{-1}.

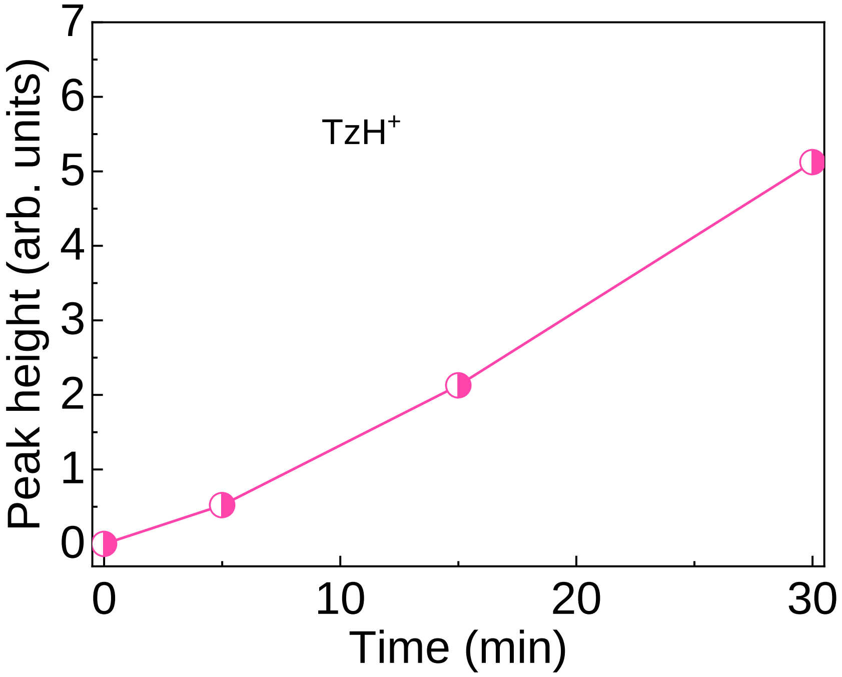

Figure S44. Peak height for TzH^+ in the in-situ FTIR spectra of COF-N32.

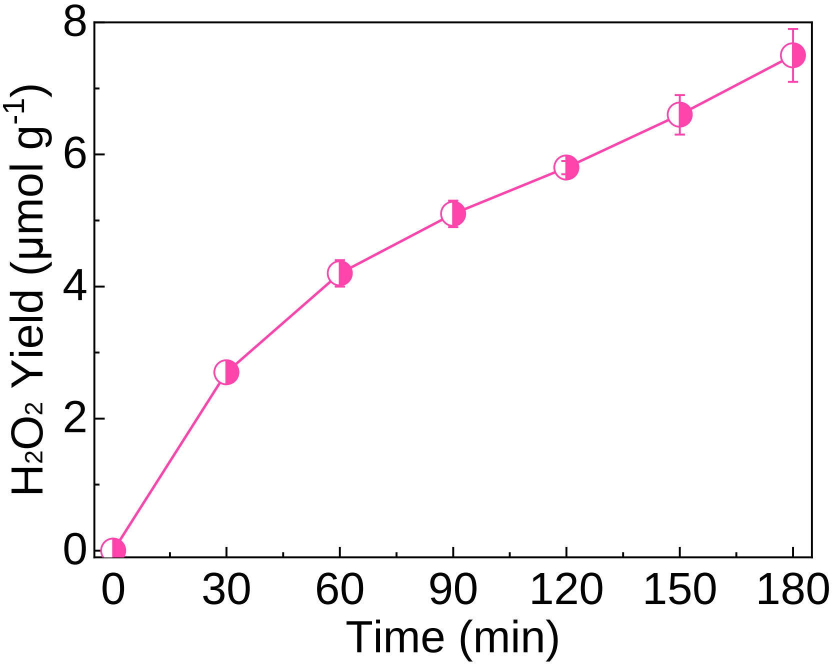

Figure S45. H_2O_2 production by COF-N32 in the presence of $NaBrO_3$ (10 mM) under N_2 atmosphere, which was determined by $Ce(SO_4)_2$ titration. Conditions: λ>420 nm (298K; xenon lamp, light intensity:100 mW·cm^{-2}), ultrapure water (50 mL). Error bars in (a) represent the average values (mean ± s.d., *n*=3).

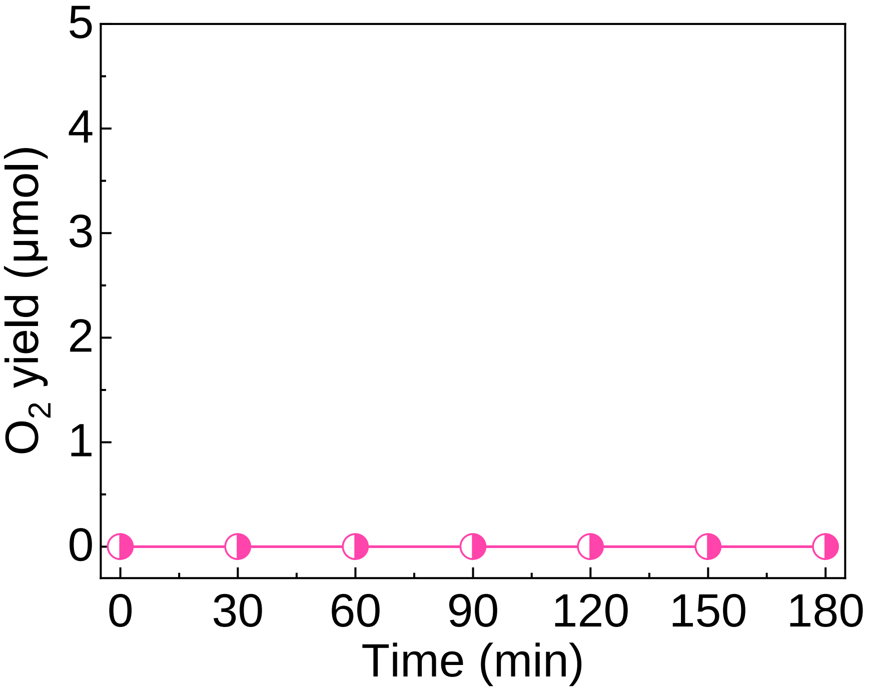

Figure S46. The oxygen evolution by COF-N32 in the presence of $NaBrO_3$ under Ar atmosphere. Conditions: λ>420 nm (298K; xenon lamp, light intensity:100 mW·cm^{-2}), ultrapure water (50 mL).

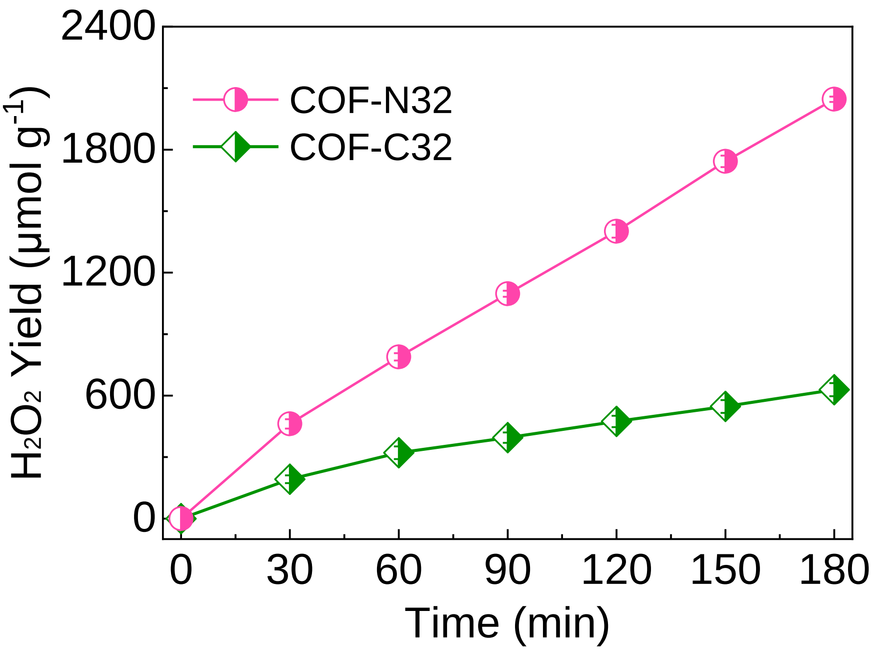

Figure S47. Comparison of H_2O_2 yield by COF-N32 and COF-C32 under visible light irradiation. Conditions: λ>420 nm (298K; xenon lamp, light intensity:100 mW·cm^{-2}), ultrapure water (50 mL). Error bars in (a) represent the average values (mean ± s.d., *n*=3).

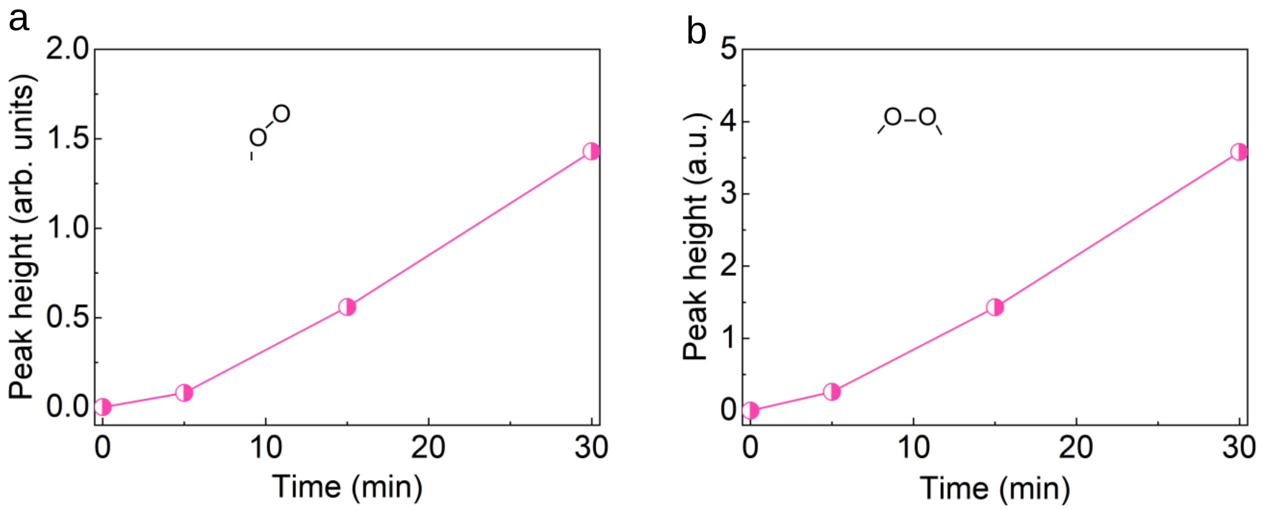

Figure S48. Peak height for (a) adsorbed $\cdot O_2^-$ and (b) peroxy species in in-situ FTIR spectra of COF-N32.

Table S1. Calculated results for intramolecular polarity of three COFs.

Photocatalysts	Dipole moment (e·Å)	Area ($Å^2$)	Intramolecular polarity ($e·Å^{-1}$)
COF-N31	3.31	46	0.072
COF-N32	6.30	198	0.032
COF-N33	8.78	440	0.020

Table S2. Calculated parameters for polarity and electrons-holes overlap of three COFs.

Photocatalysts	Molecular polarity index (MPI) (eV)	S_m index
COF-N31	0.53	0.33
COF-N32	0.50	0.36
COF-N33	0.48	0.38

Table S3. Comparison of photocatalytic H_2O_2 production in pure water by recently reported photocatalysts.

Samples	Time (hour)	Dosage (mg)	H_2O_2 yield ($\mu mol\ g^{-1}\ h^{-1}$)	Reaction conditions	Ref.
g-C_3N_4-PDI-$rGO_{0.05}$	24	50	23	Solution volume: 30 mL<br>Xe lamp (λ≥420 nm): 13 mW·cm^{-2}<br>O_2 saturated pure water<br>Temperature: 298 K	S6
RF523	24	50	51	Solution volume: 30 mL<br>Xe lamp (λ≥300 nm): 14 mW·cm^{-2}<br>O_2 saturated pure water<br>Temperature: 298 K	S7
Ag@U-g-C_3N_4-NS	1.2	100	45	Solution volume: 100 mL<br>Xe lamp (420-700 nm): 100 mW·cm^{-2}<br>O_2 saturated pure water<br>Temperature: 298 K	S8
CTF	24	30	97	Solution volume: 50 mL<br>Xe lamp (λ≥420 nm): 45 mW·cm^{-2}<br>O_2 saturated pure water<br>Temperature: 298 K	S9
Sb-SAPC15	2	100	91	Solution volume: 50 mL<br>Xe lamp (420-500 nm): 3.03 mW·cm^{-2}<br>O_2 saturated pure water<br>Temperature: 298 K	S10
ZnPPC-g-C_3N_4	--	10	114	Solution volume: 20 mL<br>Xe lamp (400-800 nm): 100 mW·cm^{-2}<br>O_2 saturated pure water<br>Temperature: 298 K	S11
TPE-AQ	1	10	909	Solution volume: 20 mL<br>Xe lamp (λ≥400 nm): 100 mW·cm^{-2}<br>Pure water<br>Temperature: --	S12

TPT-alkynyl-AQ	1	1	2030	Solution volume: 50 mL<br>Xe lamp (λ≥400 nm): 100 mW·cm^{-2}<br>Pure water<br>Temperature: 298 K	S13
CHF-DPDA	6	375	256	Solution volume: 75 mL<br>Xe lamp (λ≥420 nm): 100 mW·cm^{-2}<br>O_2 saturated pure water<br>Temperature: --	S14
RF/P3HT-1.0	6	50	237	Solution volume: 30 mL<br>Xe lamp (420-700 nm): 21 mW·cm^{-2}<br>O_2 saturated pure water<br>Temperature: 298 K	S15
Nv-C≡N-CN	1	20	137	Solution volume: 20 mL<br>Xe lamp (λ≥420 nm): 40 mW·cm^{-2}<br>O_2 saturated pure water<br>Temperature: 298 K	S16
OCN-500	10	50	106	Solution volume: 50 mL<br>Xe lamp (λ≥420 nm): 35 mW·cm^{-2}<br>O_2 saturated pure water<br>Temperature: 298 K	S17
COF-N32	3	25	702	Solution volume: 50 mL<br>Xe lamp (λ≥420 nm): 100 mW·cm^{-2}<br>O_2 saturated pure water<br>Temperature: 298 K	This work
		10	1068		
		5	1612		
		1	3168		
	12	25	605		

Supplementary References:

S1. Z. Wei *et al.*, Efficient visible-light-driven selective oxygen reduction to hydrogen peroxide by oxygen-enriched graphitic carbon nitride polymers. *Energy & Environ. Sci.* **11**, 2581-2589 (2018).

S2. M. Bhadra *et al.*, Triazine functionalized porous covalent organic framework for photo-organocatalytic E-Z isomerization of olefins. *J. Am. Chem. Soc.* **141**, 6152-6156 (2019).

S3. He, S., Rong, Q., Niu, H. & Cai, Y. Construction of a superior visible-light-driven photocatalyst based on a C_3N_4 active centre-photoelectron shift platform-electron withdrawing unit triadic structure covalent organic framework. *Chem. Commun.* **53**, 9636-9639 (2017).

S4. Liu, W. *et al.* Mechanochromic luminescent covalent organic frameworks for highly selective hydroxyl radical detection. *Chem. Commun.* **55**, 167-170 (2019).

S5. Dey, K. *et al.* Selective molecular separation by interfacially crystallized covalent organic framework thin films. *J. Am. Chem. Soc.* **139**, 13083-13091 (2017).

S6. Y. Kofuji *et al.*, Carbon nitride–aromatic diimide–graphene nanohybrids: Metal-free photocatalysts for solar-to-hydrogen peroxide energy conversion with 0.2% efficiency. *J. Am. Chem. Soc.* **138**, 10019-10025 (2016).

S7. Y. Shiraishi *et al.*, Resorcinol–formaldehyde resins as metal-free semiconductor photocatalysts for solar-to-hydrogen peroxide energy conversion. *Nat. Mater.* **18**, 985-993 (2019).

S8. J. Cai *et al.*, Crafting Mussel-Inspired Metal Nanoparticle-Decorated Ultrathin Graphitic Carbon Nitride for the Degradation of Chemical Pollutants and Production of Chemical Resources. *Adv. Mater.* **31**, 1806314 (2019).

S9. L. Chen *et al.*, Acetylene and diacetylene functionalized covalent triazine frameworks as metal-free photocatalysts for hydrogen peroxide production: A new two-electron water oxidation pathway. *Adv. Mater.* **32**, e1904433 (2020).

S10. Z. Teng *et al.*, Atomically dispersed antimony on carbon nitride for the artificial photosynthesis of hydrogen peroxide. *Nat. Catal.* **4**, 374-384 (2021).

S11. Y. Ye *et al.*, Highly efficient photosynthesis of hydrogen peroxide in ambient conditions. *Proc. Natl. Acad. Sci.* **118**, e2103964118 (2021).

S12. Y. Ye *et al.*, A solar-to-chemical conversion efficiency up to 0.26% achieved in ambient conditions. *Proc. Natl. Acad. Sci.* **118**, e2115666118 (2021).

S13. H. Yan *et al.*, Spontaneous exciton dissociation in organic photocatalyst under ambient conditions for highly efficient synthesis of hydrogen peroxide. *Proc. Natl. Acad. Sci.* **119**, e2202913119 (2022).

S14. H. Cheng *et al.*, Rational design of covalent heptazine frameworks with spatially separated redox centers for high-efficiency photocatalytic hydrogen peroxide production. *Adv. Mater.* **34**, 2107480 (2022).

S15. Y. Shiraishi, M. Matsumoto, S. Ichikawa, S. Tanaka, T. Hirai, Polythiophene-doped resorcinol–formaldehyde resin photocatalysts for solar-to-hydrogen peroxide energy conversion. *J. Am. Chem. Soc.* **143**, 12590-12599 (2021).

S16. X. Zhang *et al.*, Unraveling the dual defect sites in graphite carbon nitride for ultra-high photocatalytic H_2O_2 evolution. *Energy Environ. Sci.* **15**, 830-842 (2022).

S17. Z. Wei *et al.*, Efficient visible-light-driven selective oxygen reduction to hydrogen peroxide by oxygen-enriched graphitic carbon nitride polymers. *Energy Environ. Sci.* **11**, 2581-2589 (2018).
